# Recent advances in supramolecular fullerene chemistry†

**DOI:** 10.1039/d2cs00937d

**Published:** 2023-10-18

**Authors:** Xingmao Chang, Youzhi Xu, Max von Delius

**Affiliations:** a College of Chemistry and Molecular Sciences, Henan University Kaifeng 475004 China youzhixu@henu.edu.cn; b Institute of Organic Chemistry, Ulm University Ulm 89081 Germany max.vondelius@uni-ulm.de

## Abstract

Fullerene chemistry has come a long way since 1990, when the first bulk production of C_60_ was reported. In the past decade, progress in supramolecular chemistry has opened some remarkable and previously unexpected opportunities regarding the selective (multiple) functionalization of fullerenes and their (self)assembly into larger structures and frameworks. The purpose of this review article is to provide a comprehensive overview of these recent developments. We describe how macrocycles and cages that bind strongly to C_60_ can be used to block undesired addition patterns and thus allow the selective preparation of single-isomer addition products. We also discuss how the emergence of highly shape-persistent macrocycles has opened opportunities for the study of photoactive fullerene dyads and triads as well as the preparation of mechanically interlocked compounds. The preparation of two- or three-dimensional fullerene materials is another research area that has seen remarkable progress over the past few years. Due to the rapidly decreasing price of C_60_ and C_70_, we believe that these achievements will translate into all fields where fullerenes have traditionally (third-generation solar cells) and more recently been applied (catalysis, spintronics).

## Abstract 2

We review recent work at the interface of supramolecular chemistry and fullerene chemistry. We focus on selective fullerene functionalization, host–guest chemistry, non-covalent dyads, mechanically interlocked architectures and 2D/3D assemblies.

## Graphical abstract

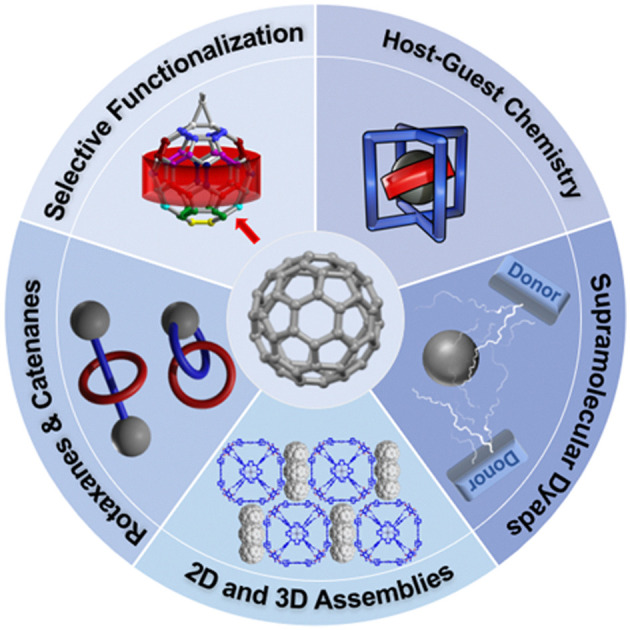

## 1. Introduction

**Fig. 1:**
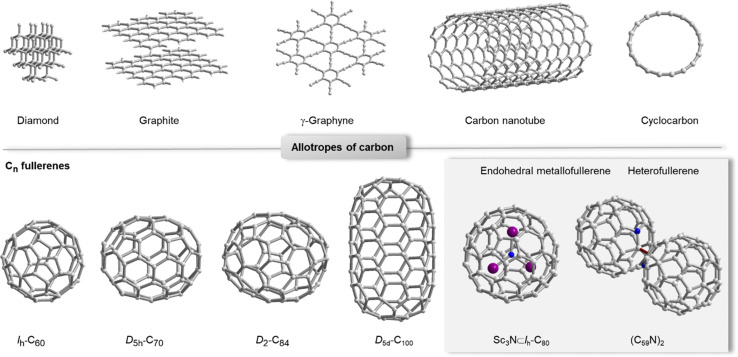
Selected examples for carbon allotropes (top row): diamond, graphite, γ-graphyne,^4^ carbon nanotube and cyclo[18]carbon.^6^ Selected examples for fullerenes C_*n*_.^12,13^ Grey box: the most abundant endohedral metallofullerene Sc_3_N⊂*I*_h_-C_80_ and heterofullerene (C_59_N)_2_ as examples for relevant compounds beyond carbon allotropes.^10,14^

Carbon allotropes come in many different forms and flavours (Fig. 1), all of which have distinct properties that are exploited in diverse functional organic materials.^1^ Diamond and graphite occur naturally and are both polymeric, but exhibit very different mechanical and electronic properties mainly as a result of sp^3^*vs.* sp^2^ hybridization of carbon. One of the unique aspects of graphene is that it can be made by exfoliation from natural graphite^2^ and by chemical bottom-up synthesis (*e.g.* by chemical vapour deposition).^3^ Synthetic carbon allotropes have seen important new arrivals such as γ-graphyne, which was prepared in bulk thanks to dynamic covalent synthesis,^4^ and a discrete, sp-hybridized member of the cyclocarbon family (cyclo[18]carbon, C18),^5^ which so far is only accessible *via* on-surface synthesis.^6^ Fullerenes^7^ and carbon nanotubes^8^ can be prepared in bulk and comprise sp^2^-hybridized carbon frameworks as well as a significant degree of curvature that influences reactivity, optoelectronic properties and non-covalent interactions. While C_60_ is the most abundant member of the fullerene family, higher fullerenes,^9^ endohedral metallofullerenes (EMFs)^10^ and heterofullerenes^11^ have unique properties due to their lower symmetry, anisotropic curvature and the incorporation of elements other than carbon (Fig. 1, grey box).

Fullerenes are engaged in a long-standing relationship with supramolecular chemistry. Concerning higher fullerenes and EMFs, this relationship is built on necessity, because their isolation from complex soot mixtures is far from trivial and requires specialized HPLC columns or innovative uses of host–guest chemistry. What makes C_60_ special as a supramolecular guest is its high symmetry (*I*_h_), relatively large size, complete rigidity, well-defined curvature and lack of heteroatoms. While its high symmetry and large size implies that sometimes C_60_ is used as a “guest of last resort” (*e.g.* in a relatively large self-assembled cage), we found that the majority of recent studies goes beyond proof-of-principle science. In this article, we therefore aim to put emphasis on advances showing exceptional originality either from the perspective of fullerene or supramolecular chemistry (or ideally: both). Because we focus mainly on advances from the past decade and we do not discuss all aspects of the field in equal detail, we wish to direct the reader to relevant work here. A book entitled “Supramolecular Chemistry of Fullerenes and Carbon Nanotubes” (edited by Martin and Nierengarten) comprises the state-of-the-art until 2012.^15^ Older review articles include overviews on metalloporphyrin hosts (2007),^16^ curved fullerene receptors (2008),^17^ open-cage fullerenes (2010),^18^ fullerene assemblies (2010)^19^ and endohedral fullerenes (2013).^10^ Among the more recent review articles, several rather narrow articles focus on methods for fullerene binding/release (2016),^20^ fullerene purification (2017, 2020)^21,22^ and selective functionalization (2020, 2021).^22,23^

It is our hope that this review article will lend further momentum to the promising research directions described herein. For the first time since 1990, two dreams of fullerene chemists have come within reach: (i) the purification of fullerene mixtures (incl. EMFs) without use of chromatography and (ii) the selective synthesis of single-isomer addition products thanks to the use of supramolecular templates. Due to the rapidly decreasing price of C_60_ (currently as low as 20 USD per gram) and C_70_, further progress towards these aims could make a real difference in key 21st century technologies, including photovoltaics, photocatalysis and quantum information processing.^24,25^

## 2. Selective fullerene functionalization

Exploiting the properties of the most abundant fullerenes C_60_ and C_70_ in solution-processed devices typically requires their covalent functionalization. Attaching one or two substituents to the fullerene core increases solubility, lowers the LUMO level, which is important for any application relying on electron transport, and modulates the morphology of solid-state materials. In this section, we will provide a brief introduction into unique challenges associated with the selective (multiple) functionalization of fullerenes and discuss supramolecular approaches towards meeting these challenges.

**Fig. 2:**
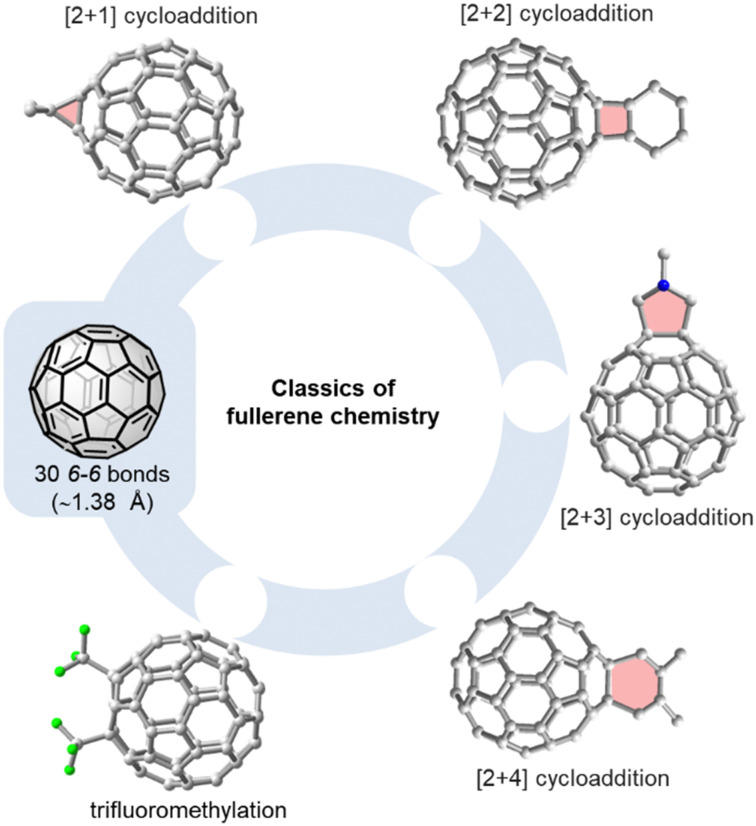
Selected methods for the functionalization of C_60_: [2+1] cycloaddition (*e.g.* Bingel–Hirsch reaction^29,30^), [2+2] cycloaddition,^31,32^ [2+3] cycoaddition (*e.g.* Prato reaction^33^), or trifluoromethylation.^34^

Phenyl-C_61_-butyric acid methyl ester (PC_61_BM) and phenyl-C_71_-butyric acid methyl ester (PC_71_BM) are typical examples of fullerene mono-adducts acting as electron acceptors or electron transport materials in bulk heterojunction or perovskite solar cells, respectively.^26,27^Fig. 2 gives an overview on the most common methods used for the functionalization of C_60_, such as the Bingel(–Hirsch) reaction, the Prato reaction, the Diels–Alder reaction and trifluoromethylation.^28^ C_70_ has a lower symmetry than C_60_ and therefore has four different types of reactive sites (α-, β-, γ-, and δ-site, see Fig. 4). This implies that even mono-adducts of C_70_ (such as the above mentioned PC_71_BM) are obtained as mixture of (regio)isomers that are hard to separate. With spherical C_60_, complex isomer mixtures are obtained whenever 2–5 groups are added to the fullerene core.

**Fig. 3:**
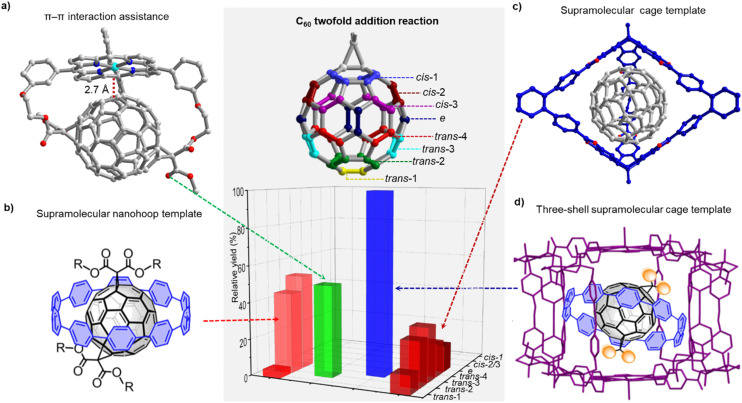
Selected methods for regioselective twofold addition reactions to C_60_. (a) π–π interaction assistance. (b) Supramolecular nanohoop template. (c) Supramolecular cage template, 3 equiv. *N*-methylglycine was used. (d) Three-shell supramolecular mask strategy.

Recent work by diverse groups in materials science established that significantly higher device efficiencies can be obtained when using isomerically pure fullerenes rather than mixtures of regioisomers, diastereomers or racemates.^23,35–37^ The classic approach to address the regioisomer problem in C_60_ bis-addition is the use of a tether. Pioneered by Diederich, this method has the disadvantage that the tether remains attached to the fullerene, thus restricting the scope of products, unless degradable linkers are used, such as the dialkoxysilanes recently employed by Nierengarten.^38–40^ Tethers can also be stimuli-responsive,^41^ and their regioisomer-directing effect can be enhanced by a non-covalent interaction, as in the example by Hirsch shown in Fig. 3a.^42^

While Kräutler's highly selective synthesis of a *trans*-1 bisadduct (96%) in the solid-state can be regarded as a supramolecular strategy,^43^ it took until 2016 for researchers to strategically employ non-covalent interactions for addressing the regioisomer problem. Torres and coworkers relied on strong intermolecular π–π interactions between two porphyrins to achieve a high regioselectivity for *cis*-substituted C_60_ bis-adducts in a Prato reaction.^44^ In 2018, our group utilized [10]CPP as a supramolecular template to synthesize [2]rotaxanes comprising a central fullerene bis-adduct as binding site for the CPP ring. The regioselectivity was not perfect, with the *trans*-1, *trans*-2 and *trans*-3 bis-adducts formed in 4%, 43% and 52% relative yield, respectively (Fig. 3b).^45^ Beuerle and Ribas independently developed the “supramolecular shadow mask” strategy that is particularly powerful, wherever a multi-addition product is desired that matches the symmetry and number of “windows” in the cage (*e.g. tris*-addition and *trans*-3 relationship between substituents in the case of the Beuerle cage shown in Fig. 3c). However, when a bis-adduct is desired, supramolecular shadow masks either give a suboptimal reaction outcome (Fig. 3c) or the reaction progress has to be stopped at precisely the right time.^22^ By combining our [10]CPP strategy and Ribas’ nanocapsule approach, we were able to achieve exclusive *trans*-3 regioselectivity for the Bingel bis-addition reaction to C_60_.^46^ This three-shell supramolecular mask strategy (Fig. 3d) required the design of an extended Pd-based cage to allow the encapsulation of the [10]CPP⊃C_60_ complex. Interestingly, the *trans*-3 C_60_ bis-adduct is symmetry-mismatched with the outer shell (three-fold *vs.* four-fold symmetry), and scope studies revealed that Rebek's 55% rule^47^ can be used to rationalize and predict the limitations of the approach.

**Fig. 4:**
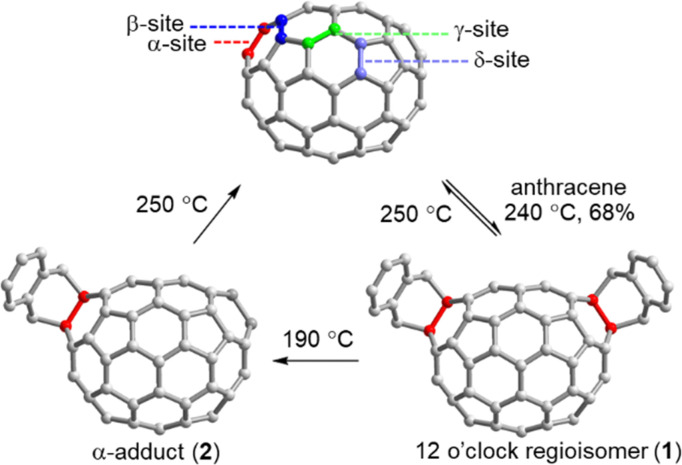
Four distinct types of double bonds in C_70_ and dynamic multi-additions of C_70_ resulting from Diels–Alder reactions in molten anthracene.

Because C_70_ has the shape of an American football, multi-adducts to this second most abundant fullerene present a particular challenge.^48–50^ Due to the decreased symmetry of the molecule eight distinct types of C–C double bonds (6,6 and 5,6), such that even mono-addition reactions give rise to isomer mixtures. While supramolecular approaches for the selective mono-functionalization of C_70_ are still elusive, Echegoyen and coworkers achieved an impressive yield of 68% for the C_70_ bis-addition 1 and obtained only one regioisomer using Kräutler's solvent-free Diels–Alder reaction process (Fig. 4). The researchers found that the C_70_ bis-adducts can be converted into the “α”-mono-adduct 2 at a temperature of 190 °C, which represents an indirect solution to the mono-addition challenge. Moreover, both mono- and bis-adducts can be reverted to pristine C_70_ at 250 °C. This anthracene addition strategy may prove beneficial as a way of protecting groups to guide multiple fullerene additions.^51^

**Fig. 5:**
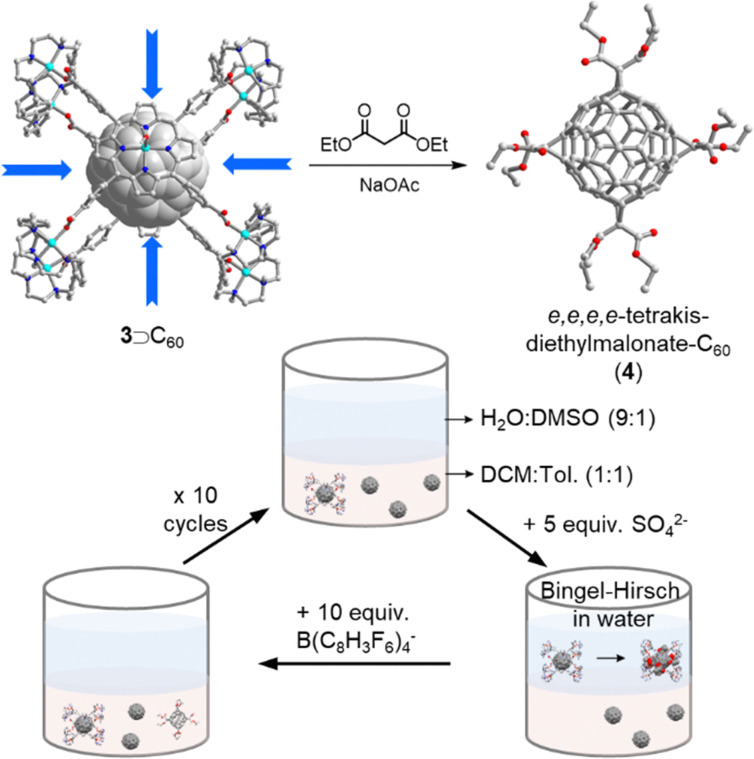
Biphasic catalytic protocol for the synthesis of *e*,*e*,*e*,*e*-tetrakis-diethylmalonate-C_60_ adduct 4 by using a sophisticated Pd-based nanocapsule 3 as the mask.

In fullerene tris- or higher addition reactions it is notoriously difficult to achieve itero- and regioselectivity. So far, only a few successful strategies have been developed based on privileged addition patterns of C_60_ encapsulated inside supramolecular masks. For instance, Beuerle and coworkers synthesized a trigonal bipyramidal covalent organic cage and observed strong affinity for C_60/70_ (*K*_a_ > 10^5^ M^−1^) in CHCl_3_. Thanks to the threefold symmetry of the cage (Fig. 3c), to the authors were able to obtain the *trans*-3–*trans*-3–*trans*-3 C_60_ Prato tris-adduct in an impressive relative yield of 25%.^52^ In a Pd-based nanocapsule shadow mask, Ribas and coworkers reported the first synthesis of an *e*,*e*,*e*,*e*-tetrakis-C_60_ Bingel adduct in quantitative yield (Fig. 5).^53^ The nanocapsule's four perpendicular “windows” led to the observed regioselectivity, and the authors were also able to achieve the selective and quantitative formation of exclusively tetrakis-adducts using a biphasic protocol and catalytic amounts of the nanocapsule. More recently, Torres, Torre and coworkers realized the efficient Diels–Alder reaction between C_60_ and anthracene in water by using a metallo–organic Pd(ii)-subphthalocyanine (SubPc) capsule as the catalytic host.^54^ In summary, supramolecular masks represent a promising new approach to achieve selective fullerene addition reactions, but atom economy is an obvious problem of this approach, because the masks typically have a higher molecular weight than the encapsulated fullerene and/or contain precious metals. It is therefore noteworthy that the masks can be recovered, and an important next goal is to develop methods that only require catalytic quantities in homogeneous solution.

**Fig. 6:**
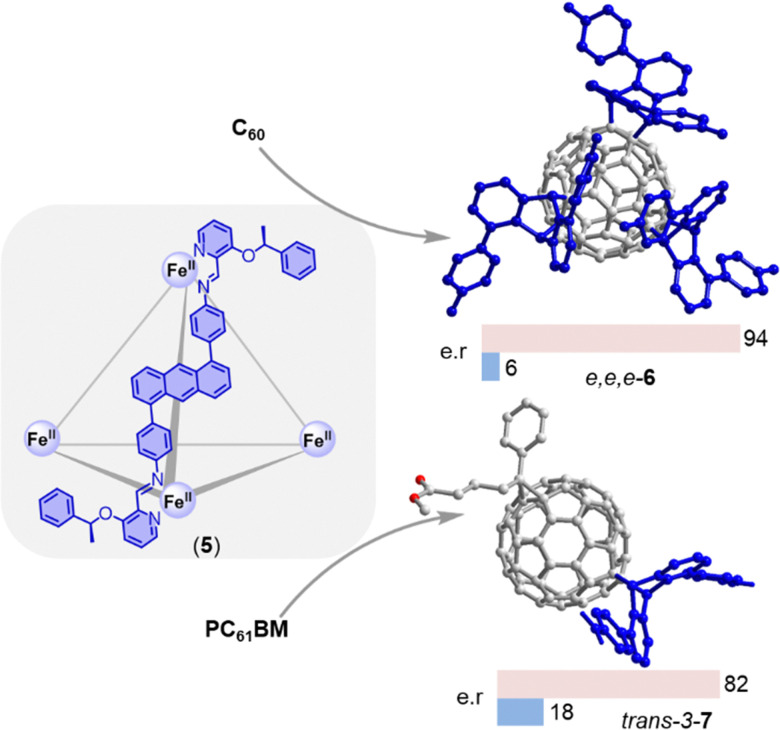
Enantioselective synthesis of an *e*,*e*,*e*-tris adduct-C_60_6 and *trans*-3-bisadduct-PC_61_BM 7 through stereochemical information transfer from a chiral self-assembled cage 5.

Because most fullerene multi-adducts are chiral, achieving enantioselectivity is a formidable challenge whose importance has been underscored by recent work by Fuchter demonstrating that homochiral fullerenes outperform the corresponding racemates as electron transport layers in perovskite solar cells.^37^ While this challenge has been approached previously using chiral reagents/catalysts,^55^ one recent report by Nitschke and coworkers demonstrated the first supramolecular approach enabling the enantioselective functionalization of fullerenes (Fig. 6).^56^ The authors utilized an enantiopure metal–organic cage (5) that was self-assembled from Fe(NTf_2_)_2_, chiral 2-formylpyridine, and 1,5-anthracene-based dianiline. The cage was shown to react with encapsulated C_60_ to produce a highly diastereoselective *e*,*e*,*e*-tris adduct (6) *via* a chemo-, regio-, and enantio-selective Diels–Alder cycloaddition with the anthracene component of the cage. Encouraged by the successful diastereoselective reaction observed with C_60_, the researchers investigated the reaction with PC_61_BM. They found that PC_61_BM reacted with only one of the six anthracene components on the opposite hemisphere to the original substituent, resulting in an adduct (7) that exhibited excellent diastereoselectivity for *trans*-3 addition. The chiral adducts produced by this method were released from the cage by adding excess tris(2-aminoethyl)amine, which led to disintegration of the cage. The chiroptical properties of these adducts were also investigated, which make them promising materials for chiral organic electronics.^57^ While these findings represent a significant advance in the field of enantioselective fullerene functionalization, a method that facilitates enantioselective addition reactions with external reagents (rather than with components of the cage) would be the next logical step.

## 3. Supramolecular fullerene dyads and triads

Electrochemical experiments show that C_60_ exhibits six equidistant reduction waves, with the lowest reduction potential at *ca.* −0.44 V (*vs.* SCE). The unique spherical and rigid framework of sp^2^ carbon atoms in C_60_ offers exceptional properties as an electron acceptor.^58,59^ It has been established that C_60_ derivatives possess relatively small reorganization energies during an electron transfer process, which makes them ideal electron acceptors for energy conversion and storage applications.^60^ In this section, we will review recent work on the electron-accepting properties of fullerenes in supramolecular dyads and triads with electron donors.

To mimic the multicomponent photosynthesis process, various covalently/non-covalently bridged C_60_-based donor–acceptor (D–A) dyads have been developed since the first pioneering work revealed that charge-recombination is considerably slower than charge-separation in porphyrin–fullerene dyads.^32,61^ The design of efficient photoinduced electron transfer systems depends on several factors, including aromaticity, planarity, and energetics of the electron donor, the distance and orientation between the donor and acceptor, and the nature of the bridging unit. Numerous C_60_-based D–A systems, containing donors such as porphyrin, ferrocene, platinum complex, tetrathiafulvalene (TTF), boron dipyrromethene (BODIPY), oligothiophene, phenothiazine or phthalocyanine have been studied to investigate the photoinduced charge separation and solar cell characteristics of these dyads.^62–65^

**Fig. 7:**
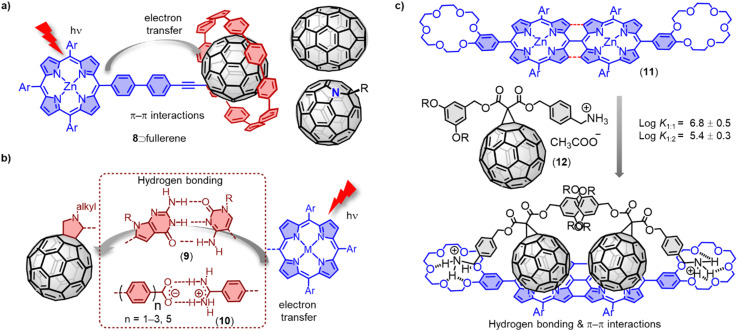
(a) Efficient electron transfer in modular 8⊃fullerene complexes. (b) Selected examples of hydrogen bond bridged C_60_-based D–A dyads (9 and 10) and corresponding charge transfer process. (c) Supramolecular complexation between ditopic porphyrin receptor 11 and fullerene derivative 12.

Although covalently bridged D–A systems based on fullerenes have shown promising results, non-covalently bridged D–A systems resemble more closely natural photosynthetic systems, particularly in respect to the bridge between donor and acceptor. A wide range of non-covalent interactions (*e.g.* π–π, electrostatic, metal–ligand, H bonds) have been employed for the construction of D–A assemblies.^66^ [10]Cycloparaphenylene ([10]CPP) exhibits a strong supramolecular association with fullerenes due to concave–convex π–π interactions.^67^ Our group has used [10]CPP as a supramolecular junction to create modular dyads between zinc porphyrin and five representative fullerenes (Fig. 7a).^68^ Fluorescence titrations revealed that all fullerene derivatives have a remarkably high association constant with 8 (*K*_a_ > 10^5^ M^−1^). Time-resolved transient absorption studies showed efficient charge separation and recombination across the non-covalent [10]CPP junction. This efficient supramolecular connection allowed studying the electron-accepting properties of the rather unstable dimer (C_60_)_2_, and interesting stoichiometry and concentration dependent effects on charge recombination were observed. For example, desymmetrization of the C_60_ moieties resulted in two distinct charge recombination processes in the 1 : 1 complex. While only one electron-recombination process was observed in the more symmetric 2 : 1 complex, a lifetime of up to 542 ns was observed, which is among the longest-lived charge-separated states in porphyrin–fullerene containing D–A systems found to date.

Hydrogen bonding-based noncovalent interactions have been widely employed to control the electronic coupling between D–A dyads. For instance, Hirsch and coworkers have reported the assembly of a C_60_-based Hamilton receptor and metalloporphyrin-based cyanuric acid motifs.^69^ Sessler and coworkers have reported zinc porphyrin-appended cytidine and fullerene-appended guanosine (9).^70^ In these supramolecular wires, the photoinduced charge transfer processes have occurred exclusively through the hydrogen bonding bridges. The attenuation factor (*β*) is a key parameter of a molecular bridge that determines the magnitude of the electronic coupling between the redox sites and the energy of the charge transfer states localized at the two ends. Martín and colleagues have reported a series of noncovalent C_60_-based hybrids (10), which combine a zinc porphyrin with *p*-(2-fulleropyrrolidinyl)benzoates of different lengths (Fig. 7b).^71^ The authors achieved an exceptionally small *β* value of *ca.* 0.07 Å^−1^, benefitting from the strong supramolecular interactions in the carboxylate/amidinium salt bridge. Nierengarten and coworkers have investigated the supramolecular complexation of a C_60_ derivative 12 (Fig. 7c) with a porphyrin dimer and a porphyrin tape (11) endowed with two crown ether rings.^72,73^ Both ditopic porphyrin systems formed complexes with 1 : 1 and 1 : 2 stoichiometry and exhibit negative cooperativity, indicating a reduced binding constant for the complex of the second fullerene unit. The formation of these complexes is driven by the complementary π–π interactions and ammonium-crown ether hydrogen bonding interactions between the porphyrin tape and the C_60_ moieties.

**Fig. 8:**
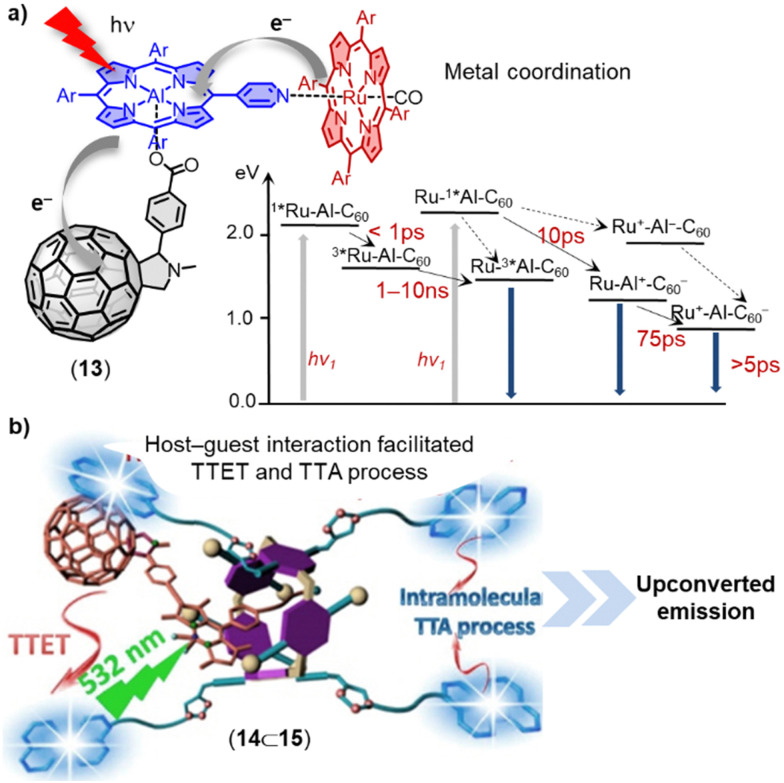
(a) Self-assembled ruthenium(ii)porphyrin–aluminium(iii)porphyrin-fullerene triad 13. Insert: Photophysical processes occurring in the triad (in CH_2_Cl_2_). (b) Schematic illustration of host–guest complexation facilitated triplet–triplet annihilation upconversion (TTA-UC) of 15⊃14. Adapted from ref. 88 with permission from American Chemical Society, copyright 2016.

The use of fullerenes and carbon nanotubes to mimic natural photosynthesis in supramolecular multicomponent D–A assemblies *via* metal–ligand coordination is another promising field of research.^74,75^ Porphyrins and phthalocyanines have been extensively employed as electron donors in the construction of fullerene-based dyads/triads due to their exceptional photophysical and photochemical properties, as well as their capacity to form metal–ligand coordination bonds.^76–79^ For instance, Lengo and coworkers effectively fabricated a three-component multichromophoric assembly 13, which consists of a fullerene monoadduct, an aluminium(iii)-monopyridylporphyrin, and a ruthenium(ii)-tetraphenylporphyrin (Fig. 8a).^80^ The photophysical properties of this triad have been examined on the femtosecond–nanosecond timescale using pump–probe spectroscopy. Upon excitation of the aluminium(iii)-monopyridylporphyrin, the strong emission characteristic of this moiety was quenched. The transient absorption experiments provided evidence for the occurrence of stepwise photoinduced electron and hole transfer processes, resulting in the formation of a charge-separated state between the fullerene acceptor and the ruthenium-porphyrin donor (see Fig. 8a for the Jablonski diagram). Architectures of higher complexity such as tetrads, pentads, and hexads have also shown promise for photoinduced electron-transfer processes.^81–84^ The non-covalent assembly between multiple fullerenes or chromophores is another promising approach for preparing organic photosensitizers.^85–87^

The process of triplet–triplet annihilation upconversion (TTA-UC) is of interest for efficiently harvesting diffuse visible/near-IR light and achieving high upconversion quantum yields.^89^ To improve TTA-UC efficiency, Yang and coworkers developed a new strategy using host–guest complexation between an alkyl nitrile chain functionalized C_60_-BODIPY sensitizer (14) and a tetraperylene-based pillar[5]arene emitter (15) (Fig. 8b).^88^ This supramolecular complexation facilitated triplet–triplet energy transfer (TTET) and TTA processes between the sensitizer and emitter, resulting in a significant increase in TTA-UC intensity and a high upconversion quantum yield (*Φ*_UC_) of up to 3.2% even at a very low emitter concentration of 6 × 10^−5^ M. This innovative supramolecular approach for bringing several components into spatial proximity improves TTA-UC efficiency without altering the intrinsic photophysical properties of the sensitizers and emitters.

**Fig. 9:**
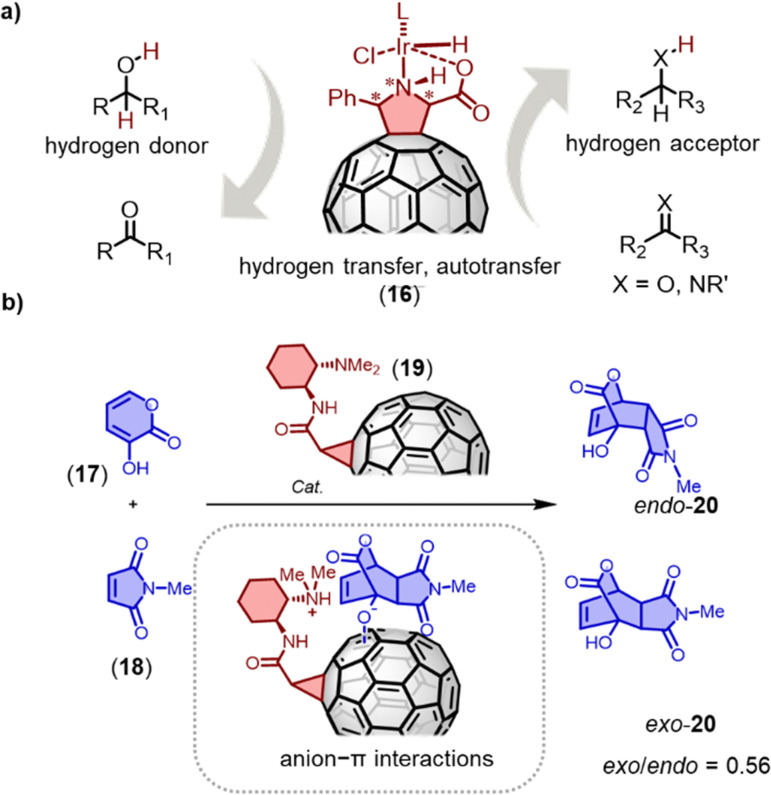
(a) Transfer hydrogenation by metallo-fulleropyrrolidine 16. (b) Anionic Diels–Alder reaction of 17 and 18; insert: proposed anionic transition state for *exo* products on C_60_ surfaces.

Fullerene-based materials are being increasingly recognized for their electrophilic nature and their ability to stabilize radicals, which makes suitable C_60_ derivatives promising in the field of (photo)catalysis.^66,90^ For instance, Heredia and coworkers have synthesized a novel boron pyrrol hydrazine-C_60_ (BOPHY-C_60_) dyad that not only produces singlet oxygen (^1^O_2_) and superoxide radical anion (O_2_˙^−^) under irradiation with visible light (470 nm), but also demonstrates the ability to photoinactivate microorganisms.^91^ Similarly, Martín and coworkers have employed metallo-fulleropyrrolidines as homogeneous/heterogeneous catalysts for hydrogen transfer reactions (Fig. 9a), which resulted in a quantitative yield of ketone reduction and alcohol *N*-alkylation with only 0.5 mol% and 0.125 mol% iridiumfulleropyrrolidine catalyst 16 loading, respectively.^92^ The catalyst is easily separable from the reaction mixture and has also been successfully utilized for the alkylation of aniline with aliphatic alcohols in the presence of MgSO_4_. For example, benzylamine underwent quantitative alkylation with cyclohexanol using a 1.25% iridiumfulleropyrrolidine catalyst.

Anion–π catalysis involves stabilizing anionic transition states and intermediates through anion–π interactions on aromatic surfaces.^93,94^ Fullerenes, which offer a highly symmetric π system devoid of heteroatoms, provide an excellent platform to investigate the importance of polarizability in anion–π catalysis, without the complications of substituents, positive quadrupole moments or in-plane dipoles.^95^ Matile and coworkers discovered that a fullerene mono-adduct catalyst 19 exhibited good selectivity for enolate addition and improved the *exo*/*endo* diastereoselectivity of the Diels–Alder reaction.^96^ Using the anionic [4+2] cycloaddition of dienophile 18 and 3-hydroxy-2-pyrones 17 as an example, strong *endo* selectivity was observed. Fullerenes with flexible tethers failed to alter this intrinsic selectivity. The best *exo*-**20**/*endo*-**20** ratio of 0.56 with 55% ee was achieved using fullerene 19 with conformationally constrained tethers as catalysts. The experimental data suggests that anion–π stabilization persists during the subsequent charge delocalization in the intrinsically disfavored *exo* transition state of the [4+2] cycloaddition.

## 4. Fullerene host–guest chemistry

### 4.1. Fullerenes as hosts

Open-cage fullerenes with their extremely rigid, all-carbon backbones exhibit unique cavities for the binding of neutral or charged guests.^10,97–100^ Although endohedral metallofullerenes (EMFs) do not meet the definition of a supramolecular complex but rather of a carceplex,^101^ we will also discuss this compound class, because non-covalent interactions in open-cage intermediates are relevant during statistical or rational EMF syntheses.

**Fig. 10:**
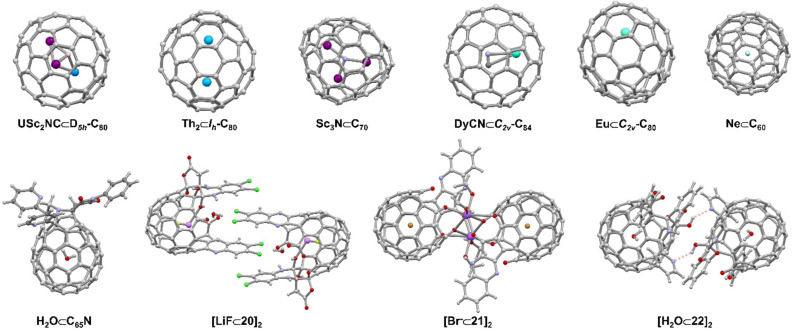
Selected endohedral fullerenes and open-cage fullerenes acting as hosts^104,109,112,114,119,121^ and supramolecular open cage fullerene-based dimers.^122–125^

Since the first report of a metal-encapsulating fullerene in 1985,^102^ endohedral fullerenes have been studied extensively.^97–99,103^ Endohedral fullerenes can be classified according to their “imprisoned” guests as (i) endohedral metallofullerenes (EMFs) and (ii) nonmetal endohedral fullerenes.^10^ The first category includes mono-, di-, and trimetallofullerenes,^104–109^ as well as cluster metallofullerenes^110^ (*e.g.* oxide clusters,^103^ nitride clusters,^111–113^ cyano clusters,^114^ sulfide clusters, and carbide clusters),^10,97–99,115–120^ while the second category includes H_2_, noble gases,^121^ water and other non-metal guests.^10,100^ (Fig. 10) The synthesis of endohedral fullerenes is a challenging task, for which several strategies have been developed, including the vaporization of graphite in the presence of additives, the implantation of atoms through the walls of the pre-existing carbon framework, as well as multi-step chemical synthesis involving the opening of orifices in the fullerene scaffold.

The first open-cage fullerene was synthesized by using singlet oxygen to oxidize an azafullerene.^126^ Pioneering work by a number of research groups^18,127,128^ led to a wide scope of open-cage fullerenes, which offer the possibility to encapsulation and entrap guests within their cavity. The encapsulation of H_2_, O_2_, CO_2_, NH_3_, CH_4_, HF, HCN, HCCH, CH_3_OH, and H_2_O was achieved in this way and progress in this field has been reviewed recently by Gan.^127,129^ In addition to the neutral molecules listed above, halide anions, LiF ([LiF⊂20]_2_, Fig. 10), and cationic [BeF]^+^ have also been encapsulated in the fullerene cage by Gan and coworkers.^123,124^ The authors demonstrated that a 19-membered open-cage fullerene with four carbonyl groups, an ether oxygen and a quinoxaline moiety on the rim of the orifice could act as the container for F^−^, Cl^−^, Br^−^ ([Br⊂21]_2_, Fig. 10) and I^−^. Fullerenes containing halide anions exhibit higher polarity compared to the respective empty fullerenes. The functional groups attached to open-cage fullerenes, such as amines, alcohols, and aromatic moieties, not only enable the selective recognition of guests, but also provide possibilities for further assembling higher-order structures or enabling the study of fundamental guest properties. For example, hydrogen-bonded fullerene dimers were obtained utilizing amide-^125^ ([H_2_O⊂22]_2_, Fig. 10) and bis(hemiketal)-^130^ containing open-cage[60]fullerenes. Dimers were also found in other work due to coordination between metal ions (such as Na, Ag, and Pt) and donor atoms as well as intermolecular π–π interactions.^123,124,131–133^ Murata and coworkers constructed a supramolecular complex by encapsulating a ^3^O_2_ molecule into an open-cage C_60_ derivative, of which the EPR spectra exhibited triplet state character as well as the anisotropy of the ^3^O_2_.^134^

Endohedral fullerenes, while technically not host–guest complexes, nevertheless offer a unique opportunity to study metal–fullerene interactions,^135,136^ unusual metal–metal interactions,^107,109,116,137^ and other special bonds within fullerenes. These effects can give rise to single-molecule magnetism.^138–141^ For example, lanthanide dimetallofullerenes featuring a single-electron Ln–Ln bond behave like single-molecule magnets and are therefore potential qubits for molecule-based quantum computing.^99^ Recently, Popov and coworkers discovered that anionic metallofullerenes can react with the Umemoto reagent II, resulting in the addition of CF_3_ groups to fullerenes, indicating electrophilic trifluoromethylation might be a useful method to derivate fullerenes.^142^ By employing this approach, M_2_⊂C_80_(CF_3_) (M = Tb, Y) monoadducts were synthesized, of which Tb_2_⊂C_80_(CF_3_) exhibits robust and remarkable magnetic properties with magnetic hysteresis up to 27 K. Apart from encapsulation of metals by larger fullerenes, Shinohara successfully obtained crystalline C_60_-based metallofullerenes Gd⊂C_60_(CF_3_)_5_ and La⊂C_60_(CF_3_)_5_ from their CS_2_ solution by vapor diffusion, in which CF_3_ groups highly improved the stability of these metallofullerenes.^143^ The electronic properties of endohedral fullerenes are tunable by the insertion of different metals or metal clusters.^144,145^ For instance, Lu_3_N⊂C_80_, a typical structure within the trimetallic nitride template (TNT) family, has a lower oxidation potential than C_60_ and exhibits high stability compared to other EMFs. This property makes it an ideal electron donor, and in 2019, Martin and coworkers used Lu_3_N⊂C_80_ in combination with C_60_ as an acceptor to construct an “all-fullerene” donor–acceptor system.^146^

### 4.2. Molecular tweezers as fullerene hosts

**Fig. 11:**
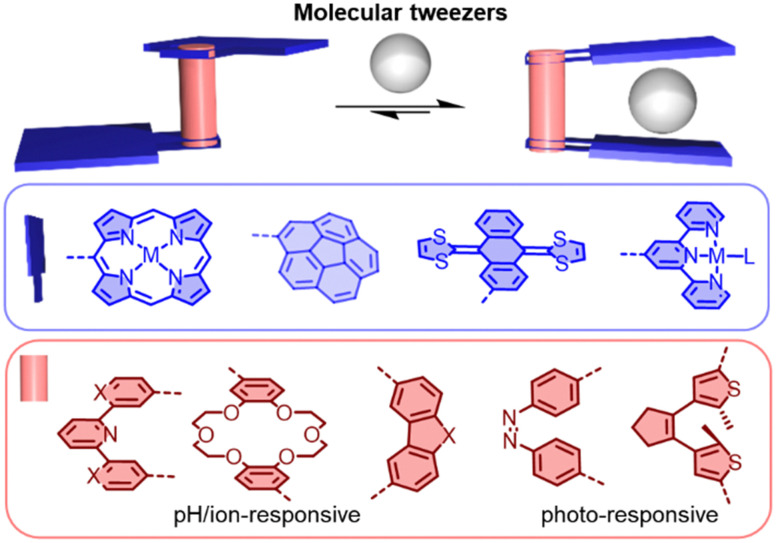
Schematic representation of switchable molecular tweezers (based on various linkers and binding motifs).

Molecular tweezers are molecular hosts with an open cavity and two identical binding sites for capturing guests (Fig. 11). By controlling the balance between rigidity and flexibility in the tweezers, distinct advantages can be provided for molecular recognition.^147^ Generally, the two binding sites can be bridged by a pH-responsive (*e.g.* pyridyl), ion-responsive (*e.g.* dipyridyl and crown ether), or photo-responsive (*e.g.* dithienylethene and azobenzene) linker, allowing the molecular tweezers to capture and release guests.^148–150^ This controllable recognition property has made molecular tweezers attractive tools in sensing, drug delivery and mixture separation. In the field of fullerene chemistry, tweezers have been studied for selectively extracting C_60_/C_70_ or other higher fullerenes from carbon soot.^151–164^

Porphyrins have a strong affinity for fullerenes, thanks to their large π surface and the capability to undergo charge transfer interactions. For instance, Boyd and colleagues devised molecular tweezers for binding C_60_ and C_70_, using porphyrin units connected *via* a metal coordination bond.^165,166^ Álvarez and colleagues synthesized double-tweezers with a single porphyrin core containing eight pyrene units, capable of binding with C_60_ (and C_70_) and forming a 1 : 2 complex. Interestingly, the double-tweezers can transform into single-tweezers by coordinating the porphyrin core with Zn^2+^.^167^ Meanwhile, π-extended tetrathiafulvalene (exTTF), with its concave geometry and high electron-donating capacity, has been extensively utilized for binding fullerenes and carbon nanotubes.^168,169^ Notably, Martín and colleagues have shown that exTTF-based molecular tweezers can be covalently linked to carbon nanotubes. By binding C_60_ relatively strongly (log *K*_a_ ≈ 3.0–3.1), interesting non-covalent C_60_/CNT hybrid materials can be obtained.^170^

**Fig. 12:**
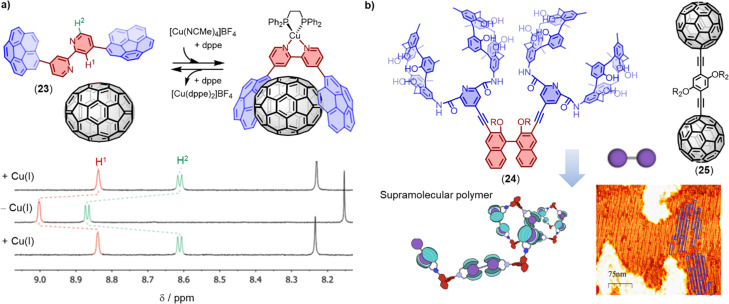
(a) Chemical structure of 2,2′-bipyridine-bridged molecular tweezers 23 and its *in situ* coordination/decoordination of C_70_ as monitored using ^1^H-NMR in CD_2_Cl_2_ at 298 K. Adapted from ref. 176 with permission from Royal Society of Chemistry, Copyright 2021. (b) Schematic representation of the formation of supramolecular polymers based on biscalix[5]arenes tweezers 24 and dumbbell fullerenes 25. Adapted from ref. 177 with permission from American Chemical Society, copyright 2021.

Corannulene is a bowl-shaped polycyclic aromatic hydrocarbon (PAH), which is predisposed for the binding fullerenes, because it represents a fragment of C_60_. In recent years, several π-extended and N-embedded “buckybowls” have been synthesized.^151^ These buckybowls exhibit high affinity towards fullerenes through concave–convex π–π interactions when incorporated in tweezers architectures.^171,172^ Sygula and coworkers provided the first strong evidence of supramolecular binding between a corannulene-based molecular tweezers and C_60_ in solution and in the solid state. The authors found that this double concave host strongly binds to C_60_ to form a stable complex (*K*_a_ = 8600 M^−1^ in tolulene-*d*_8_).^173^ Stuparu and coworkers designed a family of corannulene-based amphiphilic polymers that can yield fullerene-rich water-soluble materials.^174,175^ Shinokubo and coworkers reported two types of azabuckybowl-based molecular tweezers that exhibited different affinities towards C_60_ and C_70_.^151^ The carbazole-linked tweezers preferentially binds to C_70_ over C_60_, while the phenanthrene-linked tweezers associated with C_60_ more strongly than with C_70_. Most host–guest systems can recognize guests, but releasing the guest without destroying the host structure is often a significant challenge. Alvarez and coworkers designed 2,2′-bipyridine-bridged molecular tweezers 23 that can capture and release fullerenes through *in situ* Cu(i) complexation and decomplexation. In the presence of Cu(i), the tweezers exhibited good binding affinities with C_60_ and C_70_ (*K*_a_ (C_60_) = 2 × 10^3^ M^−1^, *K*_a_ (C_70_) = 5 × 10^3^ M^−1^ (Fig. 12a), and the reversibility of the fullerene capture and release was demonstrated.^176^

In addition to PAH motifs, macrocycles can also act as binding sites in molecular tweezers. Haino and coworkers showed that biscalix[5]arenes covalently tethered together possess an ideal cavity for accommodating fullerenes. The *K*_a_ between the biscalix[5]arenes tweezers and C_60_ was found to be approximately 10^4^ M^−1^ in toluene, significantly higher than the *K*_a_ between a single calix[5]arene and C_60_.^178^ When a molecule is equipped with two or more biscalix[5]arene tweezers, as demonstrated by molecule 24, supramolecular polymers and networks can be formed simply by addition of the dumbbell fullerene 25 (Fig. 12b). These hierarchically assembled supramolecular architectures are expected to pave the way for the development of stimuli-responsive fullerene-containing polymeric materials.^177,179^

### 4.3. Macrocycles as fullerene hosts

Pedersen, Lehn, and Cram's pioneering work on macrocyclic compounds, such as crown ethers and cryptands, established the foundations of supramolecular chemistry. Since then, there have been significant advances in the design and synthesis of new macrocyclic molecules, which have in many cases led to applications in drug delivery, sensing, and chemical separation technologies. Examples include cyclodextrins,^180^ calixarenes, cucurbiturils, pillar[*n*]arenes, pagoda[*n*]arene and cyclobis(paraquat-*p*-phenylene).^181–190^ Additionally, fully conjugated macrocycles like [*n*]CPPs, cyclo-porphyrins, and [*n*]cyclo-2,8-chrysenylenes with well-defined diameters and unique radial conjugation exhibit fascinating electronic and optical properties.^191–204^ These shape-persistent macrocycles offer significant advantages in constructing 1D nanotubes, 2D networks, and 3D complexes *via* self-assembly.^205^

**Fig. 13:**
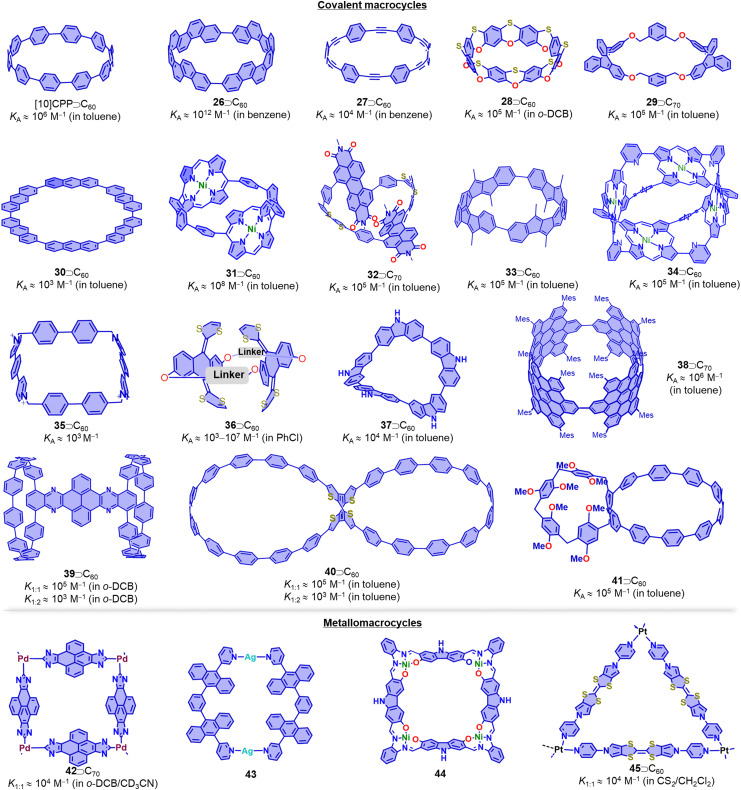
Selected fullerene hosts: covalent macrocycles and metallomacrocycles. Side-chains are omitted for clarity.

This review article primarily focusses on covalent macrocycles and metallomacrocycles, which have demonstrated their effectiveness as hosts for fullerene recognition (Fig. 13).^162,206–243^ These include highly strained and π-conjugated compounds such as [10]CPP and its derivatives,^244–251^ [4]cyclo-2,8-chrysenylene (26),^252–254^ porphyrinylene nanohoop (31),^255^ and [*n*]cyclodibenzopentalenes (33),^256,257^ which all exhibit exceptionally high affinity towards fullerenes (*K*_a_ > 10^5^ M^−1^). Among these macrocycles, [4]cyclo-2,8-chrysenylene (26) and porphyrinylene nanohoop (31) exhibit outstanding fullerene affinity (*K*_a_ > 10^8^ M^−1^) due to an extended π surface that maximizes concave–convex π–π interactions and further decreases the degrees of freedom in the host. One powerful strategy for constructing macrocyclic structures is coordination-driven self-assembly.^258,259^ For instance, Peris and coworkers synthesized a palladium-cornered metallomacrocycle 42 with four pyrene-bis(imidazolylidene) bridging ligands, which can encapsulate both C_60_ and C_70_ to form 42⊃C_60_ (*K*_a_ = 5.4 × 10^3^ M^−1^ in CD_3_CN) and 42⊃C_70_ (*K*_a_ = 7.1 × 10^4^ M^−1^ in CD_3_CN) complexes.^260^ By introducing additional homo-/hetero-macrocyclic structures to the backbone of macrocycles, *e.g.* in bismacrocycles, compounds with the capability to bind to multiple, distinct guests can be obtained.^261–266^ For example, Cong and coworkers reported a conjugated figure-of-eight oligoparaphenylene nanohoop (40) with adaptive cavities that can form 1 : 2 host–guest complexes with C_60_ and C_70_.^267^ More recently, Xu, Yam and von Delius reported the synthesis of two [*n*]cycloparaphenylene-pillar[5]arene ([*n*]CPP-P[5]A, *n* = 8 and 10) bismacrocycles by integrating P[5]A into the [*n*]CPP backbone. [*n*]CPP-P[5]A exhibits multiple guest recognition and promising properties of circularly polarized luminescence (*g*_lum_ ≈ 0.02), with [10]CPP-P[5]A showing potential for use in supramolecular polymer preparation.^268^

**Fig. 14:**
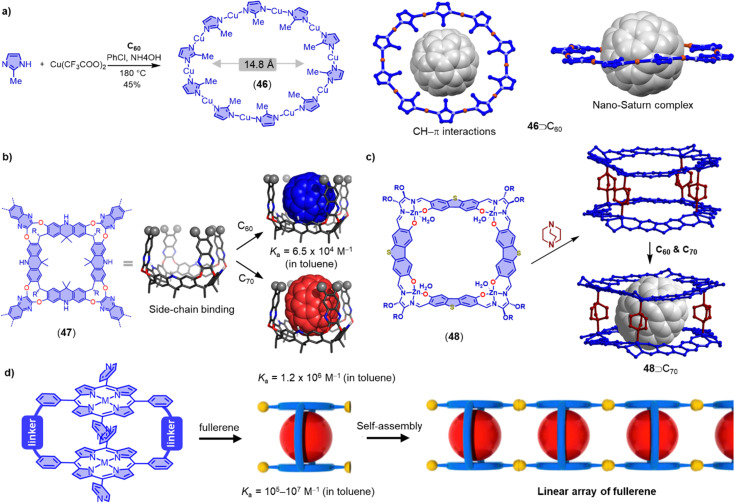
(a) Selective synthesis of [Cu_10_(2-methylimidazolate)_10_] 46 utilizing C_60_ as the template, and the solid-state structure of nano-Saturn complex 46⊃C_60_. (b) Molecular models of megalo-cavitands 47 and corresponding C_60_ and C_70_ complexes. Adapted from ref. 271 with permission from Wiley-VCH, copyright 2022. (c) Synthesis of a supramolecular double-decker cage 48 and its selectively recognition with C_70_. (d) Formation of linear array of fullerenes in self-assembled porphyrin nanotube. Adapted from ref. 272 with permission from American Chemical Society, copyright 2014.

Nano-Saturn complexes consist of a fullerene molecule as a planetary body surrounded by a macrocycle featruing a perpendicular π-system. Achieiving such complexes is not trivial, because the perpendicular arrangement of π-system limits the strength of the van der Waals (vdW) dispersion interaction. Toyota and coworkers have nevertheless demonstrated that a cyclo-2,7-anthrylene hexamer can form a disk-type nano-Saturn complex with C_60_ through multiple CH–π interactions (*K*_a_ = 2.3 × 10^3^ M^−1^ in toluene).^269^ Another promising candidate for preparing nano-Saturn complexes with fullerene is metal coordination macrocycles. However, constructing a stable and size-compatible disk-type metallomacrocycle is challenging because of the inherent lability of coordination interactions and the effects of steric crowding in the planar metallomacrocycle. Zhan and coworkers have recently reported the selective synthesis of [Cu_10_(2-methylimidazolate)_10_] 46 using C_60_ as a template.^270^ Remarkably, the ten methyl groups of the metallomacrocycle provide almost thirty CH–π interactions with the C_60_ or C_70_ molecule, stabilizing the disk-type metallomacrocycle (Fig. 14a). The interaction energy of their nano-Saturn complex is calculated to be much larger than that of most reported disk-type nano-Saturn complexes.

In addition to their direct use as fullerene hosts, macrocyclic molecules can also serve as backbones to prepare molecular cavitands, cages and nanotubes that enable fullerene encapsulation in the third dimension. For example, Tiefenbacher and coworkers have recently reported a megalo-cavitand 47 with volumes of up to 814 Å^3^ by using an acridane[4]arene and four triptycenes as building blocks (Fig. 14b).^271^ They found that 47 exhibits a higher affinity for C_70_ (*K*_a_ = 1.2 × 10^6^ M^−1^ in toluene) than C_60_ (*K*_a_ = 6.5 × 10^4^ M^−1^ in toluene). Moreover, the authors discovered that cavitand 47 can selectively bind to C_70_ in the presence of C_60_, which may be useful for fullerene purification. Tanaka and coworkers have described the synthesis of a supramolecular double-decker cage 48 composed of two shape-persistent imine-bridged tetranuclear Zn^II^-macrocycles and four 1,4-diazabicyclo-[2.2.2]octane molecules for the specific recognition of ellipsoidal fullerenes (Fig. 14c).^273^ Additionally, Tani and coworkers have designed a family of phenothiazine/alkynyl-bridged cyclic porphyrin dimers bearing self-assembling 4-pyridyl groups (Fig. 14d). The phenothiazine-bridged dimers exhibit a higher affinity with both C_60_ (*K*_a_ ≈ 10^6^ M^−1^ in toluene) and C_70_ (*K*_a_ ≈ 10^7^ M^−1^ in toluene), and these dimers can self-assemble into a nanotube through π–π interactions of the pyridyl groups and C–H⋯N hydrogen bonds between porphyrin β-CH groups and pyridyl nitrogen donors.^272,274^ Thus, macrocycle-based cavitands, macrocyclic double-deckers and self-assembled nanotubes offer opportunities for the specific recognition of fullerene derivatives.^275^

**Fig. 15:**
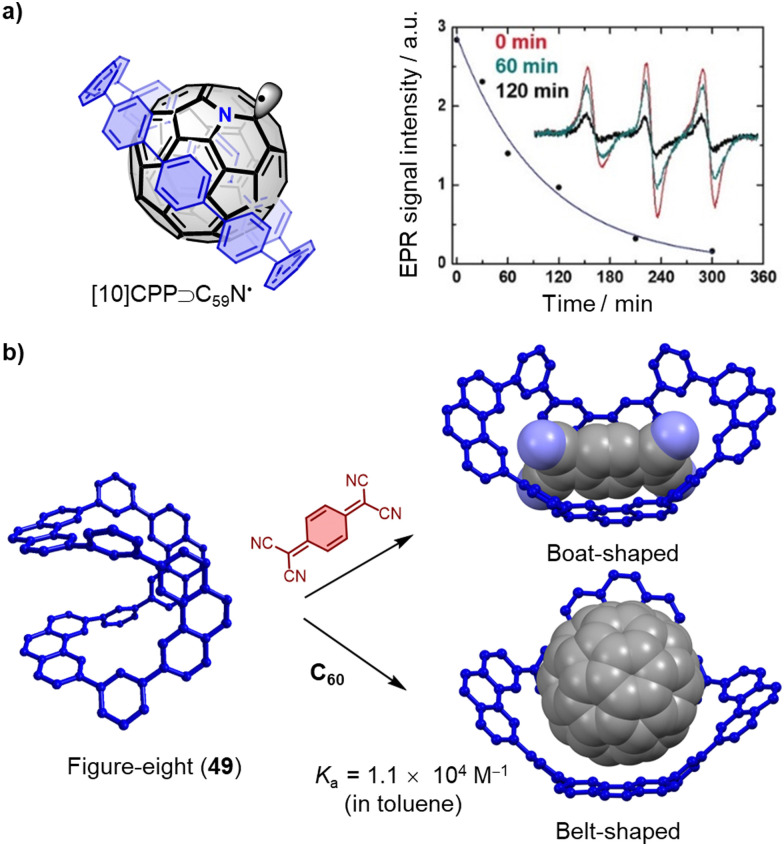
(a) Structure and time dependence of the X-band EPR signal in 1-chloronaphthalene after the illumination at 532 nm has been switched off of [10]CPP⊃C_59_N˙. Adapted from ref. 276 with permission from Wiley-VCH, copyright 2019. (b) Conformation transformation of “Figure-eight” macrocycle 49 by adding guest 7,7,8,8-tetracyanoquinodimethane and C_60_, the side-chains are omitting for clarity.

The importance of fullerene radicals in energy conversion and storage applications has been highlighted in photoinduced charge transfer processes in fullerene-based D–A systems. However, these radicals are typically short-lived and labile under air. To address this challenge, Tagmatarchis and coworkers developed a supramolecular approach to stabilize fullerene radicals.^276^ By continuously illuminating a mixture of [10]CPP⊃(C_59_N)_2_⊂[10]CPP in 1-chloronaphthalene, the highly reactive azafullerene radical (C_59_N˙) was generated and immediately shielded by formation of the stable [10]CPP⊃C_59_N˙ complex. This shielding effect leads to exceptionally long-lived C_59_N˙ radicals, which are of interest for quantum information processing technologies,^277^ because dimerization is prevented (Fig. 15a). Additionally, Tao, Du and coworkers introduced [9]CPP to the active layer of fullerene organic solar cells (OSCs). This not only promotes charge transfer between poly[4,8-bis(5-(2-ethylhexyl)thiophen-2-yl)-benzo[1,2-*b*:4,5-*b*′]dithiophene-*co*-3-fluorothieno[3,4-*b*]-thiophene-2-carboxylate] (PTB7-Th) and PC_71_BM, but also enhances charge transport between the PC_71_BM molecules by adjusting intermolecular π–π stacking. As a result, the ternary OSCs made from PTB7-Th, [9]CPP, and PC_71_BM achieved a high power conversion efficiency (PCE) of around 11%, almost one fifth higher than the PTB7-Th and PC_71_BM binary OSC devices.^278^

Conjugated macrocycles typically have rigid, inflexible cavities that restrict their ability to recognize a wide range of guest molecules and sometimes limit them to bind only a single type of guest. However, in 2021, Liu and coworkers reported a remarkable “Figure-eight” macrocycle 49, that possesses the ability to flexibly adjust its conformation in response to changes in the external environment, allowing it to accommodate a variety of guest molecules (Fig. 15b).^279^ This unique property of macrocycle 49 was demonstrated through its ability to assemble with planar, electron-deficient guest molecules, such as 7,7,8,8-tetracyanoquinodimethane, by adopting a boat-shaped conformation. The *K*_a_ of this complex was determined to be 1.0 × 10^3^ M^−1^ in toluene at 323 K. Furthermore, the macrocycle demonstrated an even higher affinity towards C_60_, with a *K*_a_ of 1.1 × 10^4^ M^−1^ in toluene at 323 K, and single crystal X-ray diffraction (SCXRD) confirmed that the macrocycle changed into a belt-shaped conformation in the presence of C_60_. These results suggest that macrocycle 49 is a promising candidate for the development of versatile host molecules capable of accommodating a diverse range of guest molecules.

**Fig. 16:**
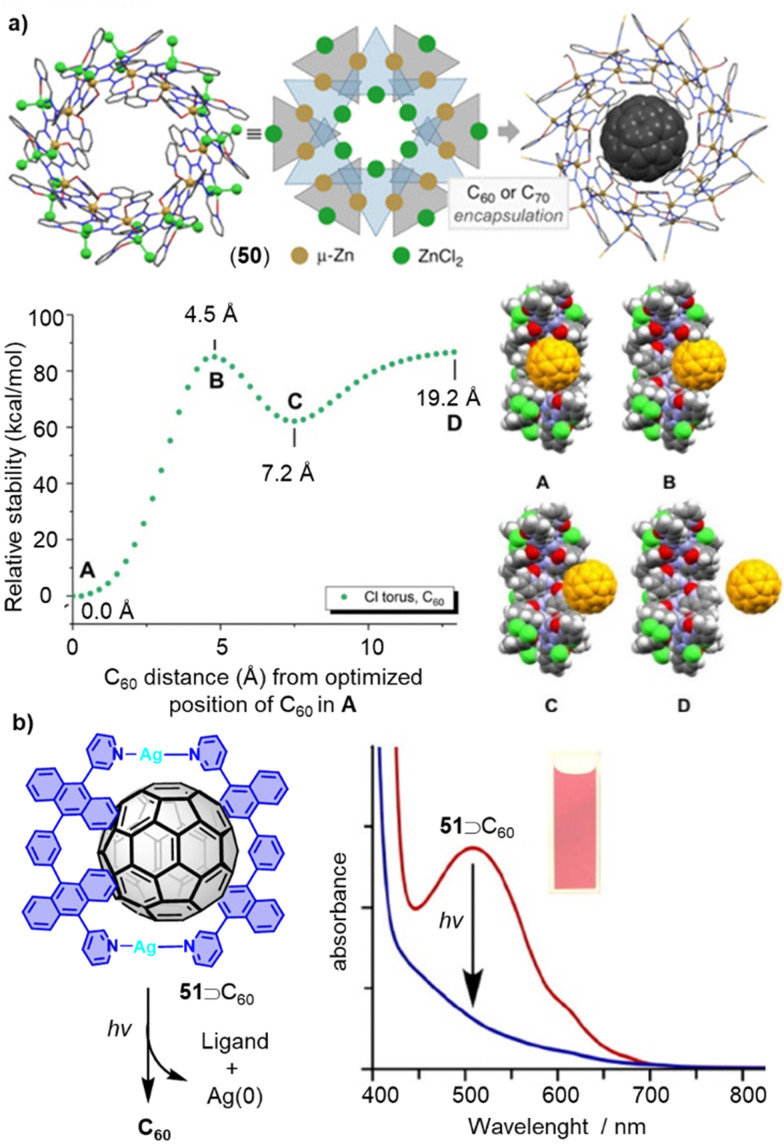
(a) Supramolecular metallocycle 50, schematic drawing and inclusion complex 51⊃C_60_ as well as energy diagram showing the relative stability as a function of the C_60_ distance from its position at the BP86/6-31G level of theory (A). Adapted from ref. 208 with permission from Wiley-VCH, copyright 2020. (b) Fullerene release from 51⊃C_60_ complex by photoirradiation. Adapted from ref. 214 with permission from American Chemical Society, copyright 2013.

Compared to covalent macrocycles, metallomacrocycles have demonstrated greater synthetic efficiency and diversity due error correction during their formation from small subcomponents. Metal coordination-driven self-assembly is a highly efficient strategy for constructing supramolecular architectures.^280,281^ Thanks to their kinetic reversibility, metallomacrocycles have wide applications in host–guest chemistry, particularly in the encapsulation and release of fullerenes.^282–285^ In 2020, Oppel reported a new torus-shaped metallomacrocycle 50 with an outer diameter of 31.7 Å. Zn(ii) ions are octahedrally coordinated between two ligands of alternating orientation which in solution, bind fullerenes C_60_ and C_70_ in their spherical cavities (Fig. 16a).^208^ The fullerene encapsulations were characterized by SCXRD and NMR spectroscopy, and theoretical calculations were conducted to gain a deep understanding of host–guest interactions in these metallomacrocycles.

For certain applications of fullerene hosts, the release of encapsulated guest molecules from the host molecule is necessary. Although many macrocycles exhibit excellent fullerene recognition ability, the subsequent release of fullerenes remains challenging, especially in cases where the binding constant is very high. Typically, guest release from host–guest complexes can be triggered by chemical, electrochemical, heat stimuli, or the addition of secondary guests in a guest–exchange process. Yoshizawa and coworkers developed a method for releasing captured C_60_ upon UV-vis irradiation (Fig. 16b).^214^ Metallomacrocycle 51 was formed only in the presence of guest C_60_. A pale-yellow solution with a powdery suspension of solids was observed after exposing 51⊃C_60_ in CH_3_CN to UV-vis irradiation produced by a 36 W incandescent light bulb at room temperature for 1.5 h. The broad UV-vis absorption band of 52⊃C_60_ (400–700 nm) disappeared, indicating the release of C_60_ from the metallomacrocycle. A recovery yield of 68% was determined for C_60_, and the complex can be regenerated in *ca.* 60% yield from the UV-vis irradiated sample by adding AgNO_3_ at room temperature.

### 4.4. Cages as fullerene hosts

Cages are a unique class of supramolecular hosts due to their three-dimensional and rigid molecular structure, typically exhibiting a well-defined cavity. In contrast to covalent or metal–organic frameworks, cages are discrete and can be characterized with solution-based techniques. Syntheses are typically achieved by self-assembly, which is why cages often feature dynamic covalent bonds,^286^ labile metal–ligand bonds^280,287^ or weak non-covalent interactions.^288^ In recent years, the encapsulation of fullerenes in cages has been pursued by a large number of groups.^16,20,289–292^ This research is typically motivated by the desire to explore new host–guest chemistry and to endow the encapsulated fullerene with unusual properties. For instance, encapsulated fullerenes can exhibit improved solubility, (radical) stability, catalytic activity or modulated redox potentials.^293–295^ As discussed in section 2, encapsulated fullerenes can also undergo itero-, regio- and stereoselective addition reactions.^52,296,297^ The selective encapsulation of fullerenes is of interest for obtaining pure fullerenes from complex mixtures (*e.g.* soot).^298–300^

**Fig. 17:**
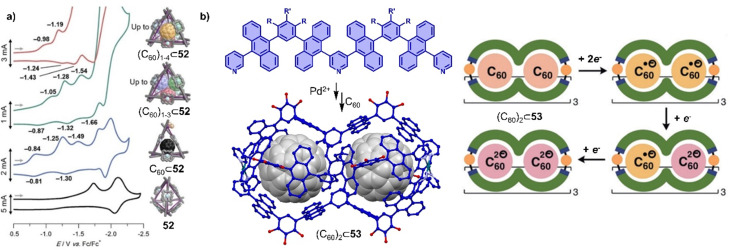
Selected redox properties of fullerenes encapsulated within the cages. (a) Cyclic voltammograms porphyrin cage 52 encapsulating 0–4 equiv. C_60_. Adapted from ref. 316 with permission from American Chemical Society, copyright 2017. (b) Assembly of peanut-shaped polyaromatic metallocage-C_60_ complex [crystal structure of (C_60_)_2_⊂53] and schematic representation of the sequential reduction processes of (C_60_)_2_⊂53. Adapted from ref. 282 with permission from Wiley-VCH, copyright 2020.

PAH moieties are often employed as the core building blocks incorporated into cages due to their strong π–π or charge transfer interactions with fullerenes. Examples of such moieties include porphyrin,^16,301,302^ pyrene,^303^ anthracene,^304^ tetrathiafulvalene,^305^ carbon bowls, as well as some electron-deficient groups like naphthalene diimide (NDI), and perylene bisimide (PBI), *etc.*^306^ Coordination-driven self-assembled cages, with diverse shapes and sizes, also known as metallocages or metal–organic cages, have been constructed and widely used for the encapsulation of fullerenes.^20,22,307,308^ The dynamic nature of metallocages, owing to labile coordination bonds (*e.g.* Pd–N) allows large fullerene guests to enter cages with small windows and transformations between different complexes,^207,302,309–312^ Some exceptionally large metallocages were shown to bind multiple fullerenes,^313^ offering an opportunity to study cluster of fullerenes.^302,314–316^ For example, a large tetrahedron metallocage 52 containing nickel(ii) porphyrins and Zn metals assembled by Nitschke and coworkers was used to encapsulate 1–4 equiv. of C_60_.^316^ Interestingly, co-encapsulation within the metallocage made it easier to reduce C_60_ to the C_60_˙^−^ radical anion, in which according to theory is due to vdW interactions between multiple fullerenes (Fig. 17a). In related work, Yoshizawa and coworkers used a peanut-shaped polyaromatic metallocage 53,^282,317^ assembled from “W” ligands and Pd(ii) metals, which allowed the encapsulation of two fullerenes separated by a distance of 6.4 Å (Fig. 17b). This discrete, non-contacted fullerene dimer undergoes sequential reduction in the cavity of the metallocage to generate (C_60_˙^−^)_2_, C_60_˙^−^·C_60_^2^˙^−^, and (C_60_^2^˙^−^)_2_. Furthermore, the stepwise encapsulation of two C_60_ molecules was achieved in a temperature-controlled fashion.

Purely organic cages have been synthesized using the well-established toolbox of dynamic covalent chemistry (DCvC).^286,318,319^ DCvC cages are typically more robust than metallocages and the products of self-assembly can be kinetically inert. The encapsulation of fullerenes into organic cages can therefore depend on the size of the cage windows, unless the cage is still dynamic, as in Beuerle's boronic ester cage.^52^ As an example for an organic cage with large windows, Zhang and coworkers synthesized a porphyrin and carbazole moieties-contained rectangular prismatic organic cage by utilizing alkyne metathesis.^320^ This cage exhibits highly selective encapsulation of C_70_ over C_60_ (*K*_C70_/*K*_C60_ > 1000), which was successfully used to isolate C_70_ from a mixture with C_60_. Of note, the encapsulated C_70_ was released by addition of excess trifluoroacetic acid and encapsulation of the fullerene was possible upon addition of triethylamine, highlighting the robustness of organic cages, which in this case enables the controlled a repeated guest encapsulation and release.

Trigonal prism-shaped cages also offer the opportunity to encapsulate fullerenes, as long as their size is in a suitable range. Recently, a trigonal prismatic nanobarrel constituted by three pyrene panels and two triangular windows with a diameter of 12.7 Å was synthesized by employing dynamic imine bond.^321^ This pyrene cage allows the encapsulation of C_60_ in poor solvents for the fullerene, which is a useful method for dissolving C_60_ in non-aromatic solvents (such as dichloromethane, chloroform, and 1,1,2,2-tetrachloroethane). In order to provide a better prediction for rational design of DCvC cages for C_60_, Jelfs and coworkers used an evolutionary algorithm to identify potential hosts for C_60_. The study showed that promising imine-based cages for encapsulation of C_60_ need to have suitable size, planar tri-topic aldehyde units with a low number of rotating single bonds, di-topic amine building blocks with functionality on adjacent carbon atoms and overall a highly symmetrical structure.^322^ Besides self-assembly based on reversible interactions, organic cages can also be synthesized by high-yielding irreversible reactions.^323^ Wu and coworkers reported a three-dimensional π-conjugated polyradicaloid prism-like cage consisting two benzene-1,3,5-triyl and three Chichibabin's hydrocarbon motifs as linker, which was prepared by Ni(COD)_2_-mediated Yamamoto homocoupling.^324^ The large cavity in the conjugated cage allowed selective encapsulation C_70_ over C_60_.

**Fig. 18:**
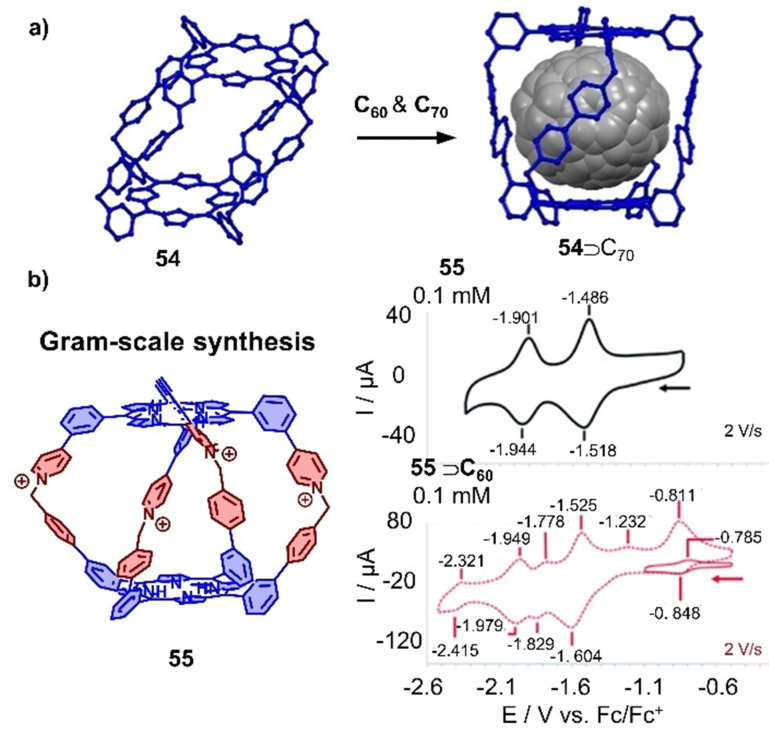
Organic cationic cages encapsulating fullerenes. (a) Selective extraction of C_70_ by a tetragonal prismatic porphyrin cage 54 (crystal structures). (b) Tetracationic cage 55 and cyclic voltammograms of 55 in comparison with C_60_⊂55. Adapted from ref. 327 with permission from Royal Society of Chemistry, copyright 2020.

A new class of organic cationic viologen/porphyrin cages was developed by Stoddart and coworkers in recent years.^325^ A tetragonal organic porphyrin cage 54 with 8 positive charges was utilized to encapsulate C_60_ or C_70_ due to the suitable cavity within cage and the favorable D–A interaction between the porphyrins and fullerene guests (Fig. 18a).^326^ More recently, Lipke and coworkers reported the gram-scale synthesis of a different cationic porphyrin cage by the formation of pyridinium linkages between two complementary porphyrin “bowls” in the last step. This new cage 55 was found to bind fullerenes very strongly (*K*_a_ > 10^8^ M^−1^ in MeCN) (Fig. 18b).^327^ Interestingly, although the cage binds C_60_ or C_70_ with strong affinities, the redox properties of fullerenes are not affected due to encapsulation, which is in contrast to other porphyrin metallocages. As both cages 54 and 55 exhibit stronger binding ability towards C_70_ over C_60_, the selective extraction of C_70_ from C_60_/C_70_ mixtures has also been achieved.

**Fig. 19:**
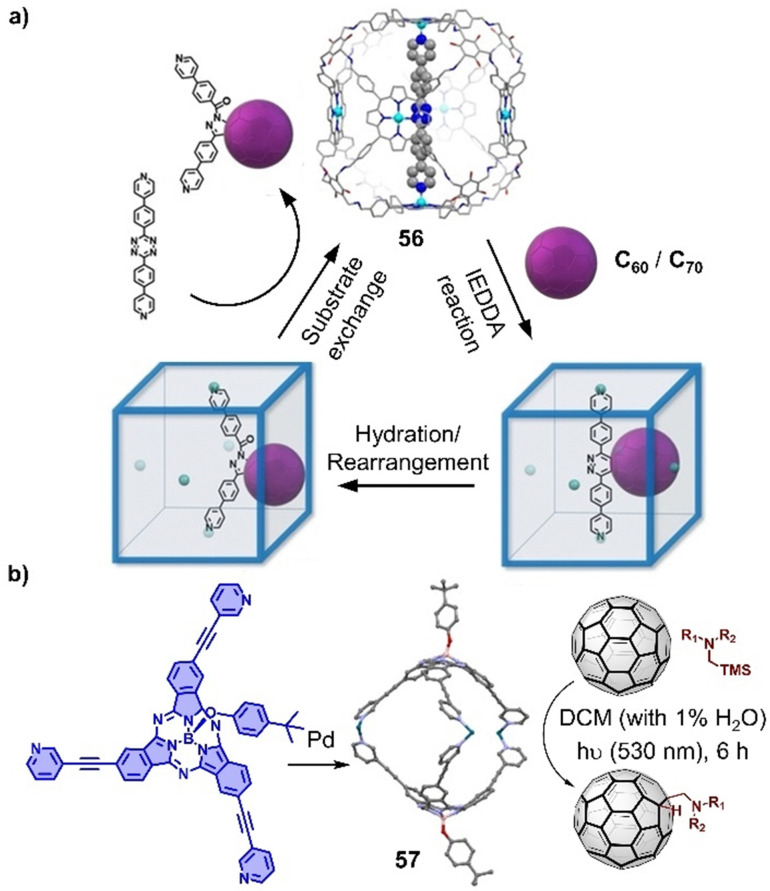
Tuning the reactivity of fullerenes within cages. (a) Representation of insertion of tetrazine-based linear axle inside a porphyrin box 56 and functionalization of C_60_/C_70_ inside box. Adapted from ref. 328 with permission from Wiley-VCH, copyright 2022. (b) Assembly of a subphthalocyanine capsule-based molecular reactor (57) and the photoredox transformations of fullerenes.

Encapsulation of fullerenes within cages has not only improved the solubility in common solvents,^321^ but also may tune the electronic properties of fullerenes due to strong π–π interactions with cage, which provide a reaction platform to modify fullerenes. Kim and coworkers constructed a series of well-defined 3D porphyrin boxes by employing dynamic covalent chemistry approach,^328^ of which the large cavity and suitable windows allow to encapsulate fullerenes and even occur those harsh condition reactions (Fig. 19a). Recently, they successfully conducted the inverse-electron-demand Diels–Alder reaction between fullerenes and 1,2,4,5-tetrazine within a Zn–porphyrin box 56, a reaction that normally requires very harsh reaction condition and longer reaction time with low yield. Interestingly, C_60_-tetrazine adduct is transferred to a bent-shaped C_60_-pyrazoline adduct through a hydration reaction and then released from the box with the addition of excess axle. The confined microenvironment within a cage also allows for photoredox chemical transformations of encapsulated fullerenes. Recently, a self-assembled subphthalocyanine capsule 57 was used as a reactor to accelerate the additions of (diaryl) methylamine and trifuoroethyl radicals to C_60_ under green light irradiation, which are typically photoredox transformations that occur within a cage (Fig. 19b).^329,330^

**Fig. 20:**
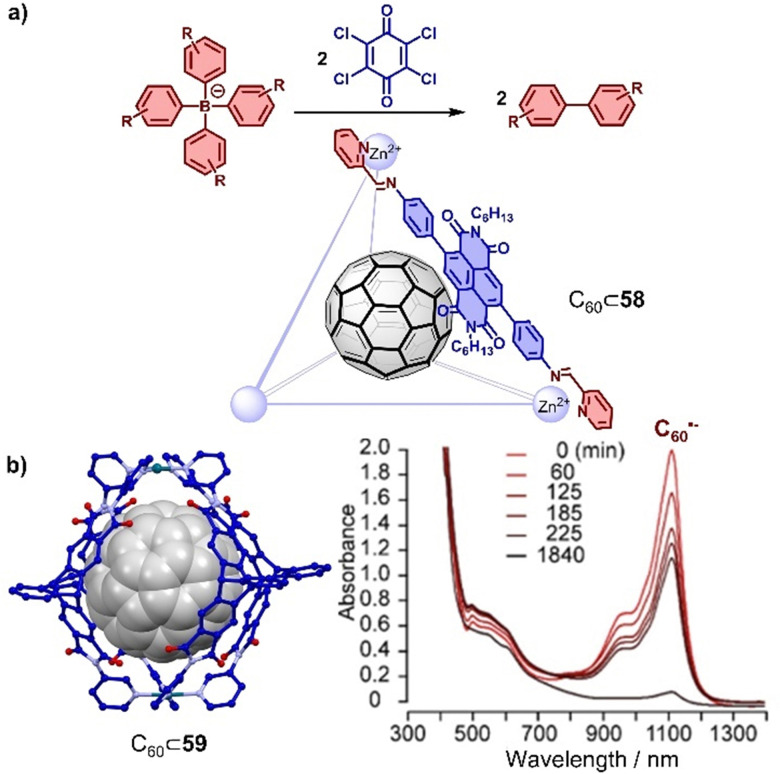
(a) C–C bond formation catalyzed by C_60_⊂58. (b) Solid state structure of C_60_⊂59 and the UV-Vis-NIR spectra of C_60_˙^−^⊂59 measured under N_2_ atmosphere over time. Adapted from ref. 294 with permission from American Chemical Society, copyright 2021.

Traditionally, electron-rich moieties are adopted as the main components to build hosts for electron-deficient fullerenes, which enhances host–guest interactions through charge transfer. However, electron-deficient moieties like PBI,^309,314^ NDI,^331^ and triptycene carboxylic dianhydride^294,332^ can also be employed to construct cages, resulting in different host–guest interactions with fullerenes. For example, Würthner and coworkers found that a Fe_4_(PBI)_6_ tetrahedron cage assembled from octahedral Fe(ii) ions and linear 2,2′-bipyridine modified PBI at the imide positions was capable of encapsulating two equivalents of C_60_.^314^ Encapsulation of C_60_ exhibits almost no effect on the absorption of cage, indicating weak interaction between PBI and C_60_ at the ground state, possibly because bulky groups at bay positions prevent the contact. Stang, Fang and coworkers also observed encapsulation of fullerenes in a PBI-based trigonal prism,^309^ assembled from Pt(ii) and tetrapyridyl connected PBI at *ortho* positions. Similar as in Würthner's study, C_60_ or C_70_ encapsulation had a negligible effect on the both absorption and fluorescence spectra of the trigonal prism, indicating that the interaction is mainly based on dispersion. The encapsulation of fullerenes in cages comprising electron deficient subcomponents offers the opportunity to stabilize fullerene or cage once either of the two is reduced to the radical anion due to the favourable interaction between acceptor and the newly formed donor (radical anion). For example, Nitschke and colleagues constructed an NDI-based Zn^II^_4_L_6_ cage 58,^331^ where the NDIs could be reduced to the radical anion and used as the catalyst for the oxidative coupling of different tetraaryl borates to give biaryls (Fig. 20a). Furthermore, the catalytic reactivity of the radical cage was further enhanced by the presence of the C_60_ guest that plays an important role in stabilizing the NDI radical. C_60_˙^−^ could act as a carrier for efficient charge harvesting and can be generated by obtaining single electrons from suitable electron donors. Generally, the lifetime of C_60_˙^−^ is rather short (<1 s) in solution. Recently, Clever and coworkers found that a self-assembled electron-deficient cage 59 consisting of four triptycene carboxylic dianhydride ligands and two Pd(ii) cations^294^ stabilized the C_60_˙^−^ radical anion, which was attributed to the strong interaction between C_60_˙^−^ and electron-deficient cage 59 (Fig. 20b). The chemical reduction of encapsulated C_60_ was achieved by adding 1-benzyl-1,4-dihydronicotinamide as a reducing regent with the 2 min irradiation of a white LED, and encapsulation which extended the half-life of C_60_˙^−^ to 14 min in air and 893 min under inert conditions.

**Fig. 21:**
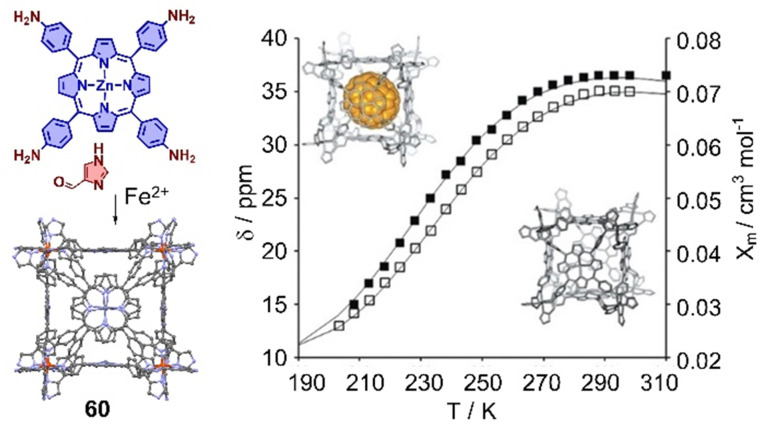
Self-assembly of an octanuclear metallocage 60 and chemical shift of selected protons in temperature-dependent ^1^H NMR experiments in CD_3_OD. Black lines represent the calculated molar susceptibility *χ*_m_ based on the ideal solution model. Adapted from ref. 333 with permission from Wiley-VCH, copyright 2017.

The interaction between cages and fullerenes not only affects their electronic properties, but also their spin state properties. For example, Lützen and coworkers reported a metallocage with spin crossover behavior, assembled by using 5,10,15,20-tetrakis(4-aminophenyl)porphyrin or its zinc(ii) complex,^333^ 1*H*-4-imidazolecarbaldehyde, and iron(ii) salts. The iron(ii) centers in this metallocage 60 exhibit the high-spin state at room temperature and low-spin state in solution at low temperature. More interestingly, a “high-spin-stabilizing effect” through encapsulation of C_70_ was observed (Fig. 21).

### 4.5. Other fullerene hosts

Hosts for fullerene binding are not just limited to tweezers, macrocycles and cages. The recent use of other types of hosts will be summarized in this section, including curved or bowl-shaped molecules,^17^ proteins,^334–336^ polymers^337,338^ and carbon nanotubes.^339,340^

Curved or bowl-shaped π-conjugated molecules are inherently more shape-complementary to fullerenes than planar compounds and possess other unique properties.^17,341–354^ For instance, many curved molecules are chiral and are more soluble than comparable planar analogues. Corannulene, which has already been discussed in the section on tweezers, is a prototypical curved π-conjugated molecule. As a fragment of C_60_, it has been a building block of choice for the binding of fullerenes.^355,356^ Dibenzo-[*a*,*g*]corannulene,^357^ a simple corannulene derivative, formed a co-crystal with C_60_ and C_70_ in 1 : 1 and 2 : 1 stoichiometry, respectively. The solid state structures indicated a strong interaction between corannulene and the fullerenes, with the shortest distance being only 3.14 Å. Shinokubo and coworkers used a nitrogen-embedded buckybowl as host for C_60_, and the 1 : 1 association of was supported by X-ray diffraction and UV-vis absorption and fluorescence titration experiments in 1,2-dichlorobenzene.^348^ A flexible decapyrrylcorannulene (DPC) host,^358^ developed by Zhang, Yang, Xie and coworkers, possesses ten pyrrole groups which resemble flexible ‘fingers’ on the periphery of the corannulene core. This host could bind 15 different fullerenes, including almost all commonly known types of fullerenes, such as pristine (C_60_, C_70_, C_90_), exohedral (six methanofullerene derivatives, three fullerene hydride derivatives, and one fulleroid derivative), endohedral (Sc_3_N@C_80_), dimeric and heteroderivatized [(C_59_N)_2_] structures, fulleroid and pentagon-fused fullerenes. Crystal structures of fullerenes with DPC reveal the high adaptivity that results from the presence of ten electron-rich ‘fingers’ at the outer rim of corannulene. Corannulene was also incorporated into organometallic complexes with the purpose of binding fullerenes. Peris and coworkers used a corannulene-functionalized di-N-heterocyclic carbene-Au^I^ complex as receptor for C_60_.^345^ The binding was found to be on the order of 10^3^ M^−1^ (toluene) and a host–guest ratio of up to 3 : 1 was confirmed by NMR spectroscopy and ITC titrations. Apart from corannulene derivatives,^359–361^ Nabeshima and colleagues developed a series of chiral concave π-systems comprising phosphorus atoms,^343,347^ which bind C_60_ due to the concave–convex interactions. In addition to buckybowls,^362–365^ a bowl-shaped nanobelt reported by Wu and coworkers, which could selectively capture C_70_ with a large binding constant (log(*K*_a_) ≈ 5; in toluene) from a mixture of C_60_/C_70_ due to size and shape complementarity.^366^ Isobe and coworkers synthesized a nanometer-sized geodesic phenylene bowl comprising 20 phenylene units, which forms a 1 : 1 ball-in-bowl complex with C_60_.^367^ The association constant in chloroform was found on the order of 10^4^ M^−1^ by ^1^H NMR spectroscopy and the complex structure was confirmed by SCXRD. Osuka, Kim and coworkers reported a series of porphyrin trimers, bearing additional carbonyl groups or methylene groups inserted between one of the β–β linkages of the porphyrin tapes. Among these hosts for C_60_, the methylene-linked *syn*-Ni(ii) porphyrin trimer exhibited the strongest association constant of *ca.* 10^7^ M^-1^ in toluene at 25 °C.^368,369^

**Fig. 22:**
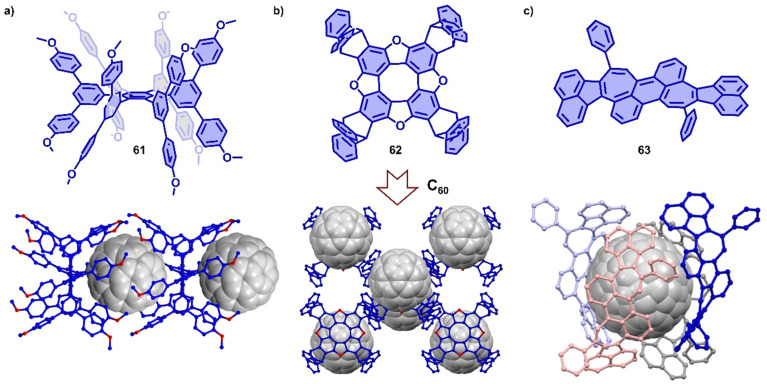
Co-crystals of Janusarene 61 (a), tetraoxa[8]circulene 62 (b) and negatively curved polycyclic aromatic hydrocarbon 63 (c) with C_60_.

π-Extended nanographenes with suitably sized cavities have also been utilized as hosts for fullerenes.^370–372^ For example, Wang and coworkers reported a Janusarene 61,^342^ possessing nineteen phenyl rings (seven constituting the hexaphenylbenzene core and the other twelve forming a “fence” around the two faces of the core), which was synthesized *via* an efficient cobalt-catalyzed cyclotrimerization of alkyne precursors. Host–guest complexation of 61 and C_60_ was supported by SCXRD, which revealed a ratio of 1 : 1 in the solid state. The crystal packing showed a 1D alternating supramolecular polymer (Fig. 22a),^373^ in which fullerene nestles inside the cavity formed by two janusarene molecules, indicating concave–convex interactions between 61 and C_60_. Wei and coworkers also reported a ‘Janus’ hexabenzocoronene derivative consisting of three triptycene units fused onto the periphery of coronene.^350^ The synthesis featured the condensation of *syn*-triveratrylbenzene and 2-formyltriptycene in one pot with high yield, and the product was found to host C_60_ (*K*_a_ = 4.9 × 10^3^ M^−1^) and C_70_ (*K*_a_ = 6.9 × 10^3^ M^−1^) in toluene. Wust and coworkers designed a series of *D*_4_-symmetric tetraoxa[8]circulenes,^374^ which possess an extended planar π-conjugation due to the introduction of oxygen atoms. One [8]circulene 62 was fused with four triptyceno subunits and could therefore serve as host for C_60_, which was demonstrated by SCXRD (Fig. 22b). Recently, Würthner and coworkers observed the co-assembly of C_60_ and a negatively curved polycyclic aromatic hydrocarbon 63, forming a supramolecular complex with an unusual 1 : 4 stoichiometry, which was characterized and analyzed by SCXRD and theoretical calculations.^375^ Interestingly, the complex topologically resembles of the fascinating carbon allotrope Schwarzite (Fig. 22c).^376^

**Fig. 23:**
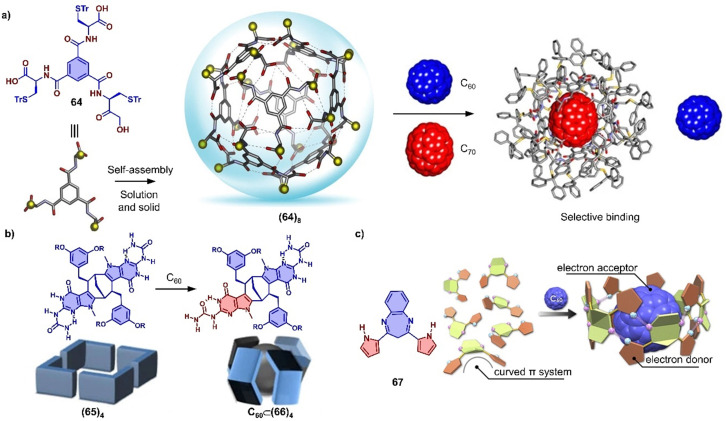
Hydrogen bond associated, adaptive systems. (a) Self-assembly of supramolecular nanocapsule (64)_8_ from compound 64 and selective encapsulation of C_70_ from a mixture of C_60_/C_70_. Adapted from ref. 384 with permission from Springer Nature, copyright 2017. (b) The symmetry breaking of monomer by rearrangement and schematic representation of starting tetramer (65)_4_ and capsular complex C_60_⊂(66)_4_ Adapted from ref. 386 with permission from Springer Nature, copyright 2017. (c) Structure of DPB 67 and schematic representation of supramolecular polymer to macrocycle transformation with addition of C_60_. Adapted from ref. 387 with permission from American Chemical Society, copyright 2020.

In addition to using large π-conjugated molecules, some small molecules also form host–guest complexes with fullerenes due to hydrogen bonding.^377–383^ For instance, Stefankiewicz and coworkers reported a hydrogen-bonded capsule, self-assembled by eight amino acid functionalized molecules 64 through 48 hydrogen bonds between the carboxyl and amine moieties of adjacent monomers (Fig. 23a), which was confirmed by SCXRD and multiple NMR methods in chlorinated solvents.^384^ The crystallographic analysis revealed that the monomer 64 formed an octameric supramolecular nanocapsule with the cavity volume up to 1719 Å^3^. The large inner cavity of capsule has been used to selectively encapsulate C_70_ from a mixture of C_60_/C_70_ in tetrachloroethane. Orentas, Wärnmark and coworkers developed a series of ureidopyrmidinone (UPy)-based hydrogen bonding tubes that exhibit intriguing dynamic behavior, such as self-sorting and solvent/guest-induced rearrangement.^380,381,385,386^ By adding C_60_, an octameric tube (65)_4_ comprising multiple hydrogen bonds was rearranged to capsular complex C_60_⊂(66)_4_ due to encapsulation of C_60_ (Fig. 23b), which was investigated by multiple NMR measurements.^386^ Recently, Maeda and coworkers reported a self-associating curved π-system,^387^ dipyrrolylbenzodiazepines (67), which forms a 1D supramolecular polymer in the solid state driven by the hydrogen bonds between a pyrrole NH as H bond donor and the diazepine nitrogen atom as H bond acceptor (Fig. 23c). The crystal structures revealed that this supramolecular polymer was successfully transformed into a large supramolecular macrocycle by the addition of C_60_, forming a supramolecular complex assembled by six compound 67 and one C_60_. The arrangement of monomer units and the strong concave–convex D–A interactions between 67 and C_60_ result in ultrafast electron transfer from 67 to C_60_.

Fullerenes are typically hosted by molecules with a rigid backbone, large π system and a suitably sized cavity. However, encapsulation of fullerenes by proteins, driven by multiple weak interactions, was also reported.^388,389^ The interactions between proteins with fullerenes and their derivatives include a very broad range, such as π–π, dispersion, hydrophobic, surfactant-like, cation–π, anion–π, hydrogen bonding, as well as electrostatic interactions, and are presumably acting synergistically,^334^ involving multiple functional groups. For instance, aliphatic moieties from amino acids like methionine, proline, alanine, leucine, valine, and isoleucine interact with the surface of C_60_,^390,391^ while amino acids with aromatic rings (*e.g.* phenylalanine) establish strong π–π stacking interactions with the fullerene.^392^ In addition, functional groups possessing positive and/or negative charges (*e.g.* lysine, aspartate and others) undergo surfactant-like interactions.^391,393^ Understanding the interaction between fullerene and protein by multiple methods (such as SCXRD, NMR, UV-vis, and fluorescence) is a complex challenge that promises insights into protein structure and dynamics.^334^

Utilizing multiple weak interactions to host fullerenes also represents a new method to construct capsules.^394,395^ Remarkably, Yoshizawa and coworkers achieved the encapsulation of fullerene in water by using a water-soluble adamantane capsule,^394^ where the driving force was the hydrophobic effect, dispersion and CH–π interactions. This approach demonstrates the potential of using weak interactions to create highly selective and efficient encapsulation systems.

## 5. Mechanically interlocked molecules

The mechanical bond^396^ is of interest for 21st century material science because it offers access to unique structural and dynamic properties as well as the ability to respond to external stimuli. Among the various types of mechanically interlocked molecules (MIMs), fullerenes are evidently good candidates as stoppers in rotaxanes, because the large and rigid carbon scaffold can prevent the dissociation of a macrocycle from a thread.^397–399^ Furthermore, fullerene-based D–A systems have been found to exhibit rapid charge separation and low reorganization energy values during photoinduced processes (see above).^400^ This unique property allows for the investigation of molecular motion and control of intramolecular photoinduced charge separation processes, which are essential steps in natural photosynthesis. As a result, the development of molecular fullerene-based MIM photosynthetic systems has gained considerable attention in recent years.^398,400^ The first fullerene-based mechanically interlocked molecule was reported in 1995 by Sauvage and colleagues,^401^ and since then, several methodologies have been developed to effectively incorporate fullerenes into pseudorotaxanes, rotaxanes, and catenanes. Metal–ligand exchange, hydrogen bonding and π–π interactionshave been instrumental for achieving these MIM syntheses.^402–404^

**Fig. 24:**
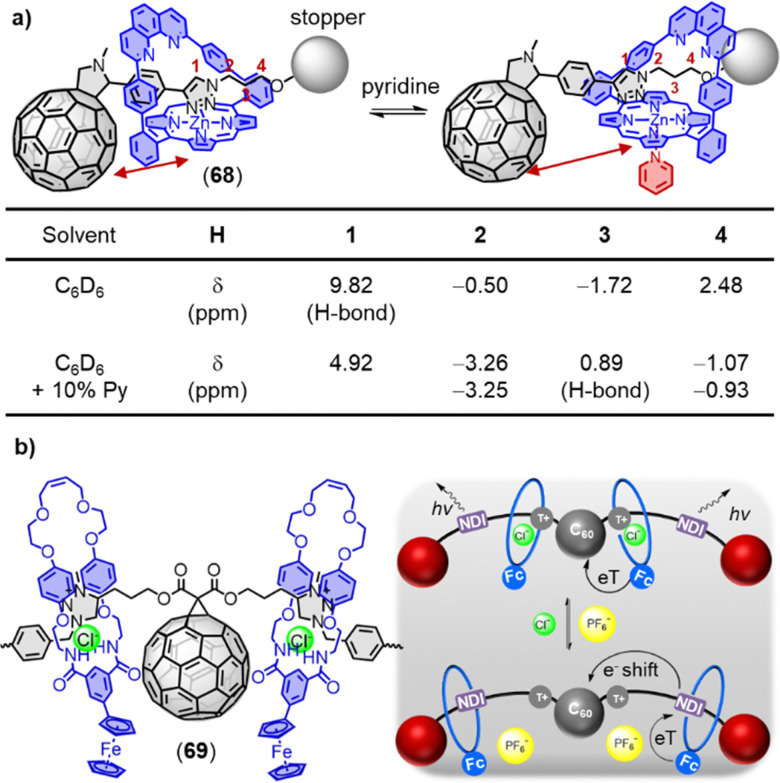
(a) Comparison of the chemical shifts and position of the dumbbell of 68 in benzene-*d*_6_ upon addition of 10% pyridine-*d*_5_. (b) Schematic depiction of a switchable D–A four-station C_60_-based [3]rotaxane 69*via* anion-induced molecular motion.

Porphyrin–fullerene MIMs are the most frequently employed in photosynthetic model systems because they reliably provide photoinduced charge separation. On many occasions, both fullerenes and porphyrins have been utilized as stoppers in rotaxanes. In 2019, Weiss and coworkers reported a novel rotaxane 68 containing a strapped porphyrin as the shuttle and a fullerene stopper at the end of a mobile dumbbell (Fig. 24a).^405^ The position of the porphyrin-based shuttle was fixed *via* metal–ligand coordination and could be switched by the addition of coordinating ligands such as 10% pyridine-*d*_5_ in benzene-*d*_6_. Pyridine binds to the Zn-center opposite to the triazole, relocating the triazole outside of the phenanthroline pocket. The motion of the shuttle was clearly supported by comparing the chemical shifts of the alkyl protons. Moreover, transient absorption measurements revealed that solvent polarity influenced molecular motion of the rotaxanes upon charge separation.

Beer and coworkers have developed a multicomponent [3]rotaxane 69 as part of their efforts to create photoactive devices with multiple switching capabilities. The rotaxane includes a four-station bis-NDI axle, a centrally positioned C_60_ bis-triazolium component, and two macrocycles containing ferrocenyl-isophthalamide. The rotaxane was prepared using a chloride anion template methodology (Fig. 24b).^406^ In the chloride form, two ferrocenyl-functionalized macrocycles reside at the center of the axle, which triggers the activation of the NDI fluorophore and formation of a C_60_ fullerene-based charge-separated state. Conversely, when the chloride anions were replaced with hexafluorophosphate anions, the ferrocenyl-functionalized macrocycles shuttle to the peripheral NDI axle stations, leading to the formation of an NDI-containing charge-separated state and quenching of the NDI emission.

**Fig. 25:**
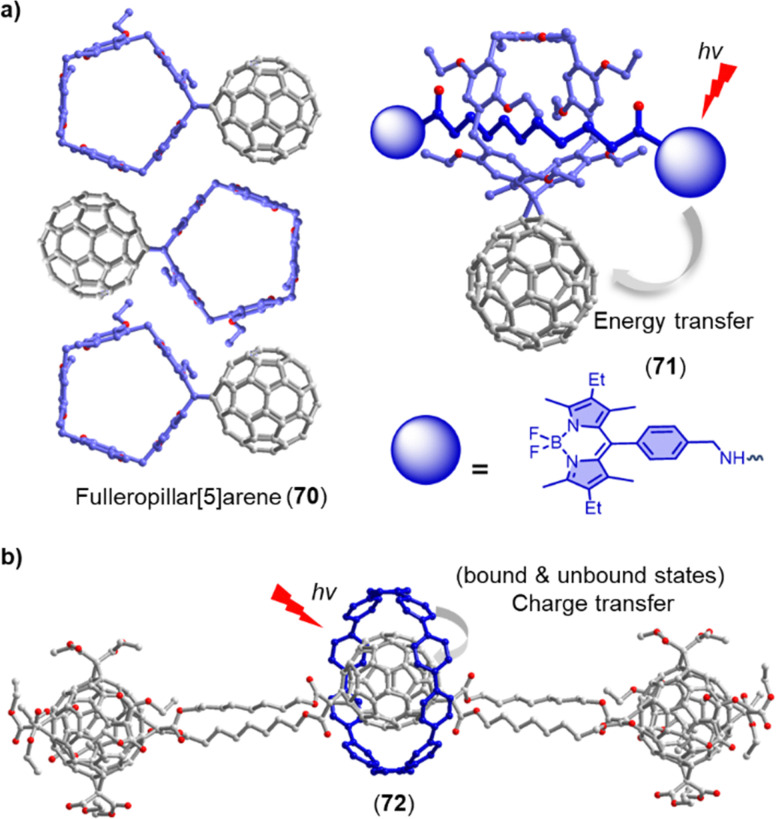
(a) Solid state packing of fulleropillar[5]arene (70) and corresponding photoactive [2]rotaxane 71. (b) Optimized structure of [10]cycloparaphenylene–fullerene [2]rotaxane 72.

Macrocyclic arenes have garnered significant attention in supramolecular chemistry due to their easy synthesis and ability to selectively recognize guests.^407^ The small, electron-rich cavity of macrocyclic arenes allows them to capture electron-deficient molecules such as alkyl nitriles, pyridinium salts, and urea derivatives, making them ideal for developing stimuli-responsive materials and constructing MIMs.^408,409^ Nierengarten and coworkers functionalized C_60_ with pillar[5]arene (P[5]A) to create a fulleropillar[5]arene derivative 70, where each fullerene moiety interacts with three P[5]A subunits through intermolecular π–π and electrostatic interactions in the solid state.^410,411^ By encapsulating a diacyl chloride in P[5]A and carrying out a stoppering reaction, the authors were able to synthesize a photoactive [2]rotaxane 71 that comprises a central fullerene moiety and two terminal BODIPY stoppers (Fig. 25a). Even though the C_60_ moiety and the BODIPY stoppers are not covalently connected, efficient through-space excited state interactions have been observed in this architecture. In 2020, Wu and coworkers reported a “fulleropillar[4]arene”: a pillararene in which one arene moiety has been replaced by a *trans*-4 fullerene bisadduct. Interestingly, this unusual macrocycle exhibits significantly higher association (*K*_a_ ≈ 6600 M^−1^ in DMSO) with viologen derivatives than P[5]A,^412^ which allowed the authors to prepare a new type of pseudorotaxane.

Constructing MIMs with large size and shape-persistent cavities, such as those found in prototypical carbon nanohoops or nanobelts, presents significant challenges, not least because these componds typically lack heteroatoms. In 2018, the von Delius group overcame this difficulty by reporting the successful synthesis of two discrete [2]rotaxanes (72) that feature a strained carbon nanoring [10]CPP, which exhibited intriguing photoinduced charge transfer properties (Fig. 25b).^45^ The strong concave–convex π–π interactions between [10]CPP and C_60_ monoadduct (*K*_a_ = 1.8 × 10^6^ M^−1^ in toluene) enabled the highly efficient and regioselective synthesis of these two rotaxanes (*trans*-2: 10% and *trans*-3: 26%). Transient absorption studies revealed snapshots of the macrocycle's “bound” and “unbound” states along the trajectory of the thread. The exceptionally slow dissociation of the CPP ring from the fullerene binding site, along with very fast translation along the thread was later confirmed in high-level meta dynamics simulations^413^ and could pave the way towards MIMs in which the ring is transporting charge, which would have relevance for photovoltaics and photocatalysis.

**Fig. 26:**
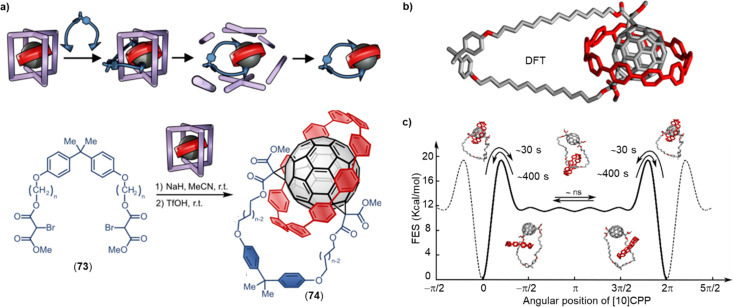
(a) The synthetic strategy of C_60_/[10]CPP-catenanes (74). (b) Atomistic molecular model of the C14-C_60_/[10]CPP-catenane. (c) Free-energy diagram as a function of the angular position of [10]CPP along the larger ring of the catenane.

Recently, the Ribas and von Delius groups used their Matryoshka approach (see Fig. 3) for the *trans*-3 selective bis-functionalization of C_60_ to achieve the synthesis of a new type of [2]catenane 74.^414^ These catenanes comprise a [10]CPP ring that is mechanically interlocked with a larger macrocycle that is formed in the final step of the synthetic route by Bingel bis-addition to C_60_. Because the malonate esters (73) are unsymmetric, this reaction could lead to an astonishingly complex reaction mixture with up to 22 spectroscopically distinguishable isomeric products. The combination of nanocapusle shadowmask (degraded in a controlled fashion after the reaction), [10]CPP template (ring incorporated into the product) and tether effect (use of bismalonates connected through a long spacer) was thus necessary to obtain >97% selectivity for *trans*-3 isomers along with *ca.* 30% yield (Fig. 26a). The authors demonstrate that for the optimal tether length (C14) an isomer fraction of up to 87% was obtained for one out of the three distinct *trans*-3 diastereomers (*in,out-trans*-3). The dynamic properties of this type of catenane were explored *in silico* by the Pavan group, who reported extremely fast translation of the [10]CPP along the non-fullerene parts of the larger macrocycle as well as a very long average residence time of 30 s on the fullerene binding site (Fig. 26b and C).

## 6. 2D and 3D assemblies

Fullerenes and their derivatives have emerged as valuable building blocks to construct functional carbon materials, which are widely used in different fields, such as sensors, liquid crystals, optoelectronics, catalysts, and energy conversion.^19,171,415,416^ To obtain highly ordered fullerene 2D or 3D fullerene materials, numerous approaches have been developed.^417,418^ While covalent cross-linking establishes robust 2D frameworks, the principles of supramolecular chemistry are vital for establishing order during synthesis and can be harnessed to generate 3D vdW (hetero)structures.

### 6.1. Assembly of ordered fullerene materials

2D carbon materials have attracted increasing attention due to their unique fundamental properties that promise applications in electronic and optical devices, as well as in charge storage, heterojunctions, hydrogenation and photovoltaic applications.^417,418^ Within the fullerene family, C_60_ has been widely used as a building block to construct 2D structures owing to its spherical shape and commercial availability. While the band gap of crystalline C_60_ is around 2.3 eV,^419^ theoretical studies predict that the band gap of both polymeric and vdW-based C_60_ monolayers ranges from 0.6 to 1.0 eV,^420^ which is significantly lower than that of any experimentally isolated monolayer of an organic semiconductor. Furthermore, the band gap of C_60_-based 2D materials highly depends on the symmetry of the structure and the distance between individual C_60_ moieties.^420,421^ Recently, some important advances have been made in preparations of highly ordered 2D fullerene-based materials, representing a new approach to synthesize 2D materials with novel (optoelectronic) properties.^422–424^ For example, Zheng and coworkers successfully synthesized covalently bonded C_60_ networks by using an organic cation slicing strategy.^422^ C_60_ molecules formed radical anions upon heating with Mg, and new C–C bonds formed between adjacent C_60_ moieties through [2+2] cycloaddition reactions. X–ray diffraction studies revealed that the bulk crystals exhibit a layered structure of alternative polymeric C_60_ layers and Mg atomic layers. The Mg layers act as linkers between the adjacent C_60_ layers by Mg–C bonds with the length of 2.22–2.37 Å, which made the C_60_ layers cannot be exfoliated by simple mechanical exfoliation.

**Fig. 27:**
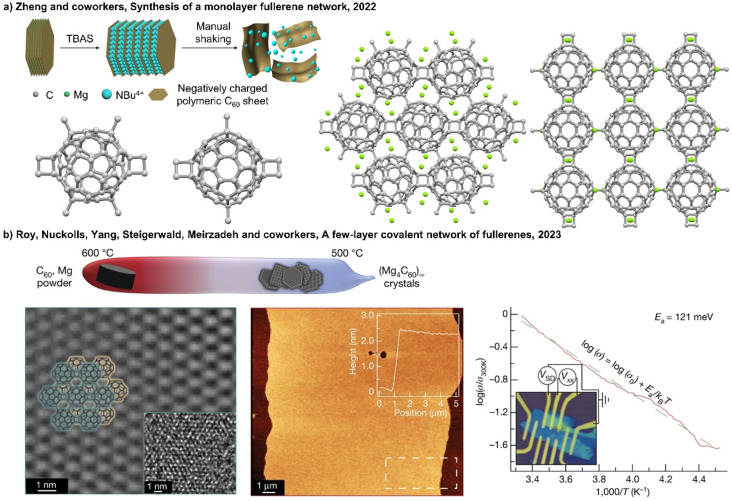
(a) Schematic of organic cation slicing exfoliation and crystal structures of the bulk single crystal of qHP-C_60_ and qTP-C_60_ as well as their monomer C_60_. Adapted from ref. 422 with permission from Springer Nature, copyright 2022. (b) Schematic of the CVT technique used for the growth of (Mg_4_C_60_)_∞_ single crystals, iFFT and AFM image of a few-layer “graphullerene” flake, the log of the conductance (*σ*) *versus* temperature (*T*) for a 70 nm-thick (Mg_4_C_60_)_∞_-based device (a fit to a thermally activated (Arrhenius) model (dashed green line), inset is a typical device and corresponding four-terminal measurement scheme). Adapted from ref. 423 with permission from Springer Nature, copyright 2023.

Thus, the authors used an organic cation slicing strategy treating the bulk crystals with tetrabutylammonium salicylate (TBAS). This led to cleavage of the Mg–C bonds and the formation of magnesium salicylate and tetrabutylammonium cations (NBu_4_^+^) that replaced the Mg cations. (Fig. 27a) The NBu_4_^+^-intercalated C_60_ crystals were subsequently exfoliated by gentle manual shaking furnishing a few-layer quasi-tetragonal phase (qTP) and a monolayer quasi-hexagonal phase (qHP). Both two 2D materials exhibit great crystallinity and unique topological structures. Compared with graphene and individual fullerene molecules, the monolayer polymeric C_60_ possesses a moderate band gap of 1.6 eV and good thermodynamic stability. The asymmetric lattice structure of the monolayer induces significant in-plane anisotropic properties, such as anisotropic phonon modes and conductivity, which can be attributed to the unique structure of the monolayer. Later in the same year, Peng rationalized the experimental band gap of these polymeric C_60_ materials *in silico*. The calculations also suggested that these monolayer C_60_ materials have suitable band gaps, charge carrier mobilities and band edges for photocatalytic water splitting.^425^

**Fig. 28:**
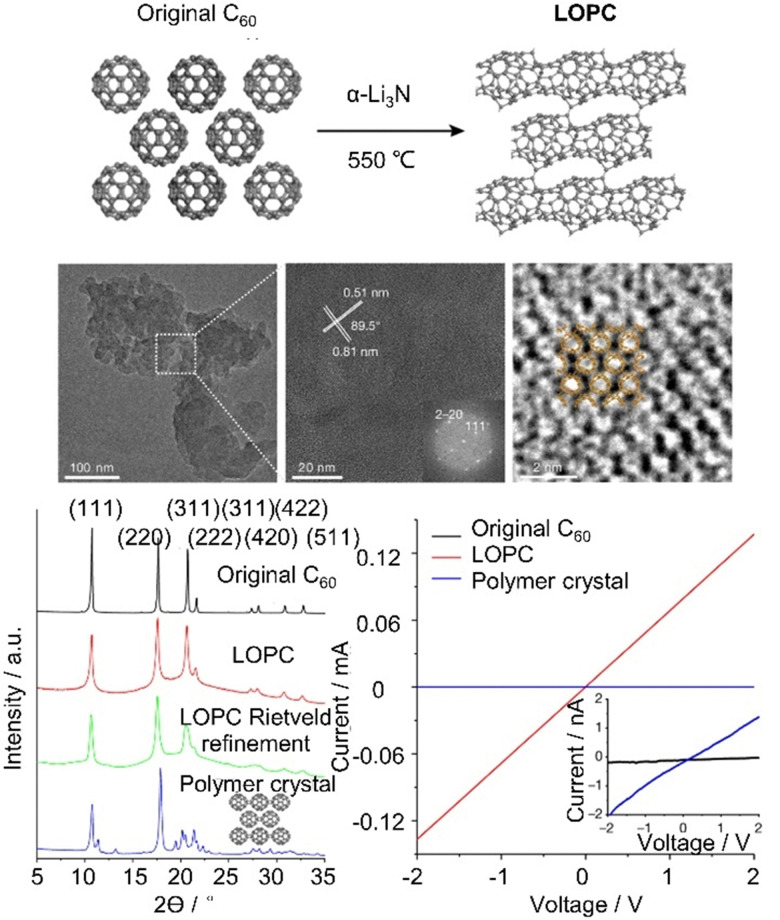
Atomic structure of LOPC, TEM images of LOPC particles, Cu Kα (*λ*  =  0.15418 nm) X-ray diffraction patterns with simulation for LOPC, and direct current voltage–current curves of three membranes made by mixing each carbon material with 5 wt%; polytetrafluoroethylene. Adapted from ref. 424 with permission from Springer Nature, copyright 2023.

Recently, Roy, Nuckolls and coworkers reported on “graphullerene”,^423^ a graphene-like hexagonal layer of polymeric C_60_ linked by covalent bonds (Fig. 27b), synthesized using the chemical vapour transport (CVT) method to grow single crystals of a (Mg_4_C_60_)_*n*_ network, followed by removal of magnesium by dilute acid. This material exhibits a much higher thermal conductivity compared to molecular C_60_, likely due to the in-plane covalent bonding of layered polymeric C_60_. In addition, Moiré-type superlattices were found by high-resolution transmission electron microscopy (HR-TEM) and near-field nano-photoluminescence spectroscopy. However, the methods used for small-scale preparation of carbon structures with covalently cross-linked C_60_ limit detailed characterization and represent a hurdle for applications. In this respect, an important advance was made in 2023 by Zhu, Ruoff and coworkers who reported the gram-scale synthesis of long-range ordered porous carbon (LOPC),^424^ a new type of carbon. The synthesis started from C_60_ and was catalyzed by α-Li_3_N at 550 °C and ambient pressure (Fig. 28). LOPC consists of broken C_60_ cages mostly bonded *via* sp^2^ carbon atoms, as evidenced by X-ray diffraction, Raman spectroscopy, magic-angle spinning solid-state NMR spectroscopy, aberration-corrected transmission electron microscopy and neutron scattering. LOPC possesses remarkable conductivity, with a value of 1.17  ×  10^−2^ S cm^−1^ at room temperature and the conduction is close to the result from a combination of metallic-like transport over short distances punctuated by carrier hopping at a temperature lower than 30 K. Simulations of LOPC reveal that graphullerene is a metastable structure that exists as intermediate during the transformation from a fullerene-type to a graphene-type carbon allotrope and represents a transition from semiconducting to metallic property with increasing temperature. Recently, Du and coworkers explored the photocatalytic water splitting properties of a few-layer C_60_ network, which furnished production rates of H_2_ and H_2_O_2_ of 91 and 116 μmol g^−1^ h^−1^, respectively.^426^ Collectively, these recent breakthroughs offer new opportunities for exploring the fundamental properties, supramolecular properties and potential applications of covalently bonded 2D carbon materials.

2D or 3D fullerene crystals are different from these covalently cross-linked 2D materials, because they rely only on non-covalent forces such as π- and vdW interactions.^19,417,418,427,428^ A large amount of 2D fullerene crystals with great diversity of shapes, such as 2D hexagons, 2D nanorhombus or hexagonal 2D nanosheets have been obtained by using the liquid–liquid interfacial participation (LLIP) method.^429–431^ For example, Ariga and coworkers obtained a new class of C_60_ crystals with bimodal pore architectures by employing LLIP with the use of solvents isopropyl alcohol (IPA), benzene and carbon tetrachloride (CCl_4_). By changing the ratio of benzene and CCl_4_, the shapes of 2D crystals were tuned. Interestingly, the bimodal pore crystals exhibited 2D hexagonal plate-like morphology and offered enhanced electrochemically active surface areas compared to pristine C_60_.^432^ Sathish and coworkers reported 2D hexagonal C_60_ nanosheets using the LLIP method at the CCl_4_/alcohol interface. The size of 2D C_60_ crystals could be tuned by changing the alcohol (anti)solvent and the diameters of hexagonal nanosheets varied from ∼7.5 μm, ∼2.5 μm, and ∼500 nm (IPA, ethanol, and methanol, respectively).^433^

**Fig. 29:**
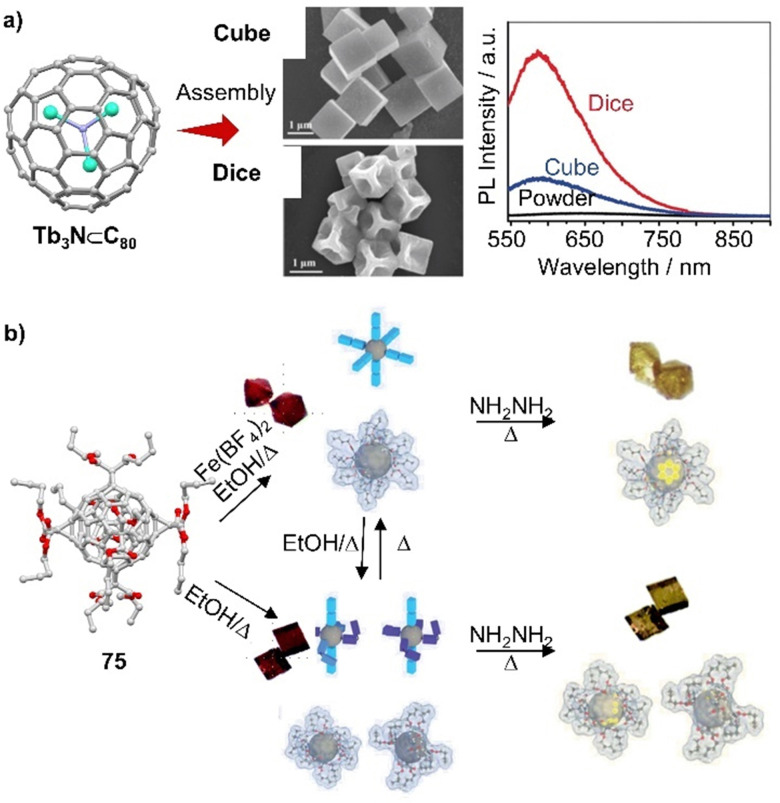
(a) SEM images of cube- and dice-shaped Tb_3_N⊂C_80_ microcrystals and fluorescence spectra of Tb_3_N⊂C_80_ dice, cube, and powder. Adapted from ref. 434 with permission from Wiley-VCH, copyright 2019. (b) Dynamic behavior of a hexakis[60]fullerene 75. Adapted from ref. 439 with permission from Royal Society of Chemistry, copyright 2021.

The synthesis of 3D fullerene crystals, which are mostly based on C_60_ or C_70_ and their derivatives, is highly dependent on the shapes of fullerenes.^434^ C_70_ 3D crystals were first prepared by Choi and coworkers in 2010 by using the precipitation approach.^435^ Since then, several other methods have been developed to obtain fullerene 3D crystals, including static liquid–liquid precipitation, re-precipitation, solvent vapor annealing, and drop-drying.^417^ When using different methods, polymorphic structures of 3D fullerene crystals can be obtained that exhibit distinct properties such as enhanced fluorescence emission, hydrophobicity and photocurrent response, high surface-to-volume ratio beneficial for electrocatalytic hydrogen evolution reaction and sensing.^417,418^ Recently, Yang and coworkers developed a universal approach based on the LLIP method associated with ultrasonication to prepare the endohedral fullerene-based 3D crystals with tunable crystal shape.^434^ Three metal nitrile “clusterfullerene” (M_3_N⊂*I*_h_-C_80_, M = Tb, Er, and Sc) 3D crystals were successfully obtained, and the shape of crystals could be easily switched by changing the solvent ratio of solvent to antisolvent. The crystal shape-dependent emission of the three clusterfullerenes was studied, revealing that crystals with dice shape emit stronger light than the cube-shaped crystals (Fig. 29a). Such an enhancement of photoluminescence in highly crystalline C_70_ and higher fullerene C_78_ relative to their powder states was also found by others, and was attributed to the increased crystallinity of fullerenes together with the decreased intermolecular interactions.^435–437^ The formation of fullerene-based 3D materials not only provides an ordered arrangement of carbon cages, but also produces a regular network of pores that are typically larger in the case of less symmetric C_70_. By changing the crystal preparation method, the porous structure can also be tuned. Shrestha, Ariga and coworkers prepared highly crystalline C_70_ cubes with the average edge lengths of *ca.* ∼3.4 ± 0.4 μm that possess holes extending 1–1.5 μm deep from the surfaces *via* dynamic LLIP.^438^ Interestingly, the holes on the surfaces of the C_70_ cube could be closed by regrowing a thin layer of fullerene, and subsequently be reopened by local irradiation *via* electron beam.

In addition to the formation of 3D crystalline materials by using pristine fullerenes, fullerene derivatives have also been utilized as building blocks to construct 3D frameworks or porous materials.^440–445^ These frameworks are assembled by non-covalent interactions, and their structures can be tuned by manipulating the dynamic interactions within frameworks. Beuerle and coworkers reported a series of hexakis-substituted C_60_ adducts bearing twelve carboxylic acid groups, and their incorporation as the polytopic organic linker into the hydrogen-bonding frameworks in solid state^446,447^ and MOFs interlinked by metal ions (such as Zn^2+^, Ca^2+^, Cu^2+^, and Cd^2+^).^448,449^ The interfullerene distances within the frameworks could be controlled by either alkyl space length between C_60_ and carboxylic acid group or the type of cross-linking.^446–449^ Recently, Martin, Costa and coworkers created dynamic molecular crystalline frameworks by using weak “sticky fingers” vdW interactions^439,450^ based on a hexakis-substituted C_60_ adduct 75 synthesized using the Bingel–Hirsch reaction (Fig. 29b). The hexakis-substituted C_60_ adduct 75 forms two different crystals from ethanol with and without Fe(BF_4_)_2_. Transformation between two polymorphs could be achieved by heating with and without ethanol. These materials are highly dynamic, such that exposure to hydrazine vapor induced the selective hydrogenation of crystalline materials and a structural change. The molecular movements in the lattice and the selective reaction can be observed directly by single-crystal to single-crystal diffraction (Fig. 29b).

Self-assembled monolayers (SAMs) have been widely used for surface modification and act as the crucial interlayers and electronically active layers in organic electronic devices (such as organic light emitting diodes, organic photovoltaics, and organic thin film transistors). Not surprisingly, C_60_-functionalized SAMs exhibit interesting properties.^451–456^ For example, Peukert and coworkers tuned the molecular order of C_60_ functionalized phosphonic acid monolayers by changing the ratio of alkyl phosphonic acids (PA) and C_60_-functionalized octadecyl phosphonic acids (C_60_-PA) on alumina substrates.^455^ A pronounced maximum in sum-frequency intensity of the C_60_ band is observed for SAMs with ∼75% C_60_-PA and ∼25% PA. By using the same method, Clark, Halik and coworkers further confirmed that a mixture of C_60_-functionalized and nonfunctionalized spacer molecules can lead to a morphology with improved charge transport in self-assembled monolayer field-effect transistors.^453^ By changing the ratio of C_60_-PA and PA from 100 : 0, 70 : 30, 50 : 50, and 30 : 70, the maximum drain currents of these monolayers were successfully tuned and quantum mechanical calculations revealed conduction pathways within the fullerene monolayers.

**Fig. 30:**
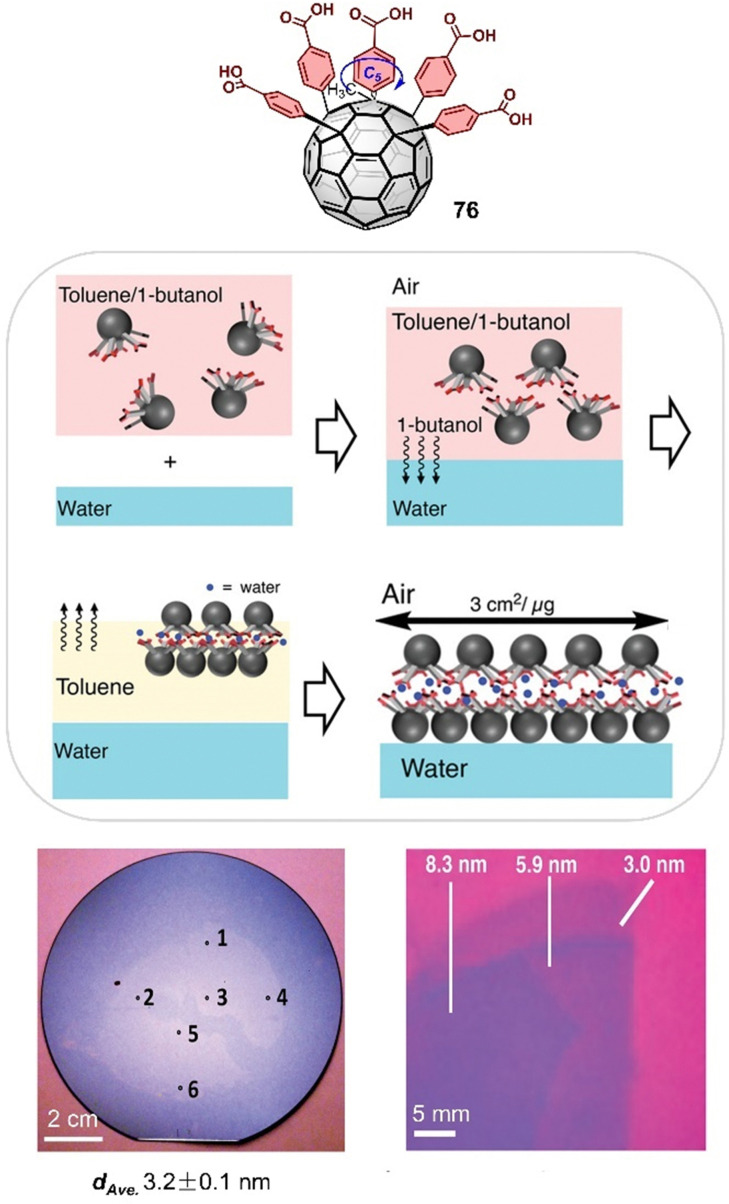
Structure of pseudo-*C*_5_ symmetric fullerene amphiphile 76, assembly of 2D film, the 2D film on 160.6 ± 0.1 nm thick SiO_2_/Si wafer (*φ* = 10.2 cm) (the thickness of six points were calculated by interference fringe shifts from visible-light reflection spectra), and sequential lamination of film on 288.8 nm thick SiO_2_/Si wafer to form single, double, and triple films. Adapted from ref. 461 with permission from Wiley-VCH, copyright 2022.

A series of fullerene amphiphiles have been synthesized,^457–459^ different from neutral derivatives, whose relevant interfacial chemistry has been summarized in 2019.^460^ Recently, Nakamura and coworkers described a pseudo-*C*_5_ symmetric fullerene amphiphile 76 attached with five 4-benzoic acid groups. The toluene/1-butanol/water solution of this compound spontaneously forms a 3 nm thick, free-standing 2D film as 1-butanol and toluene are evaporating gradually during a few hours at the water/air interface. (Fig. 30) The film was stabilized by hydrogen bonding between two fullerene layers. The size of this large-area film was up to several tens of cm^2^ and the photoconductivity of this film (transferred to a gold comb electrode) was determined as 1.4 × 10^−4^ S cm^−1^. Furthermore, the film was laminated into a multilayer film either by using large amount of 76 solution or repeating the preparation procedure several times.^461^

**Fig. 31:**
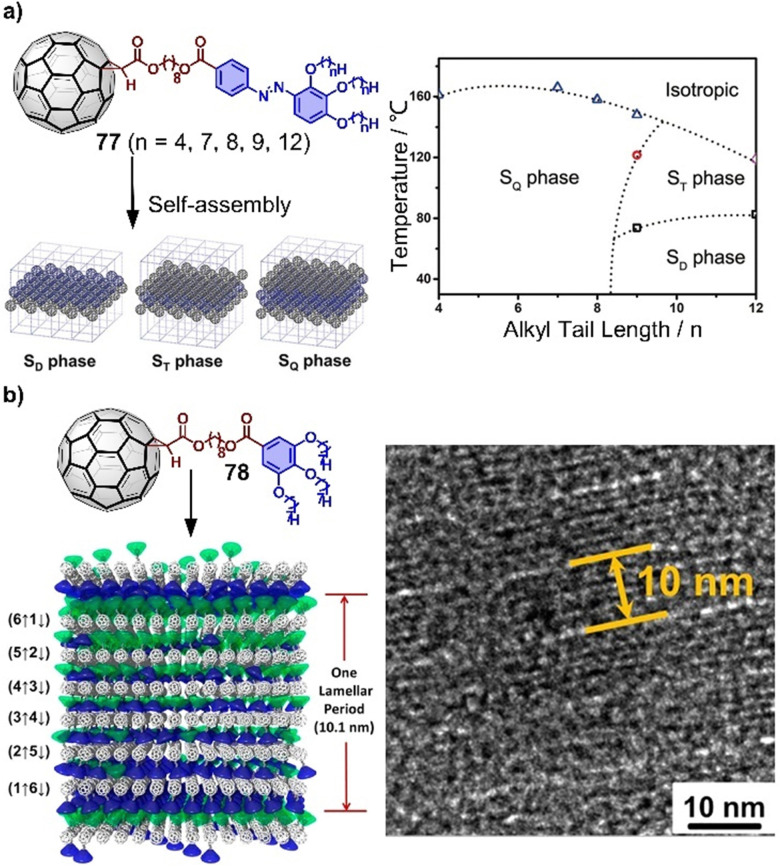
(a) Molecular structure of tetrablock-mimicazobenzene-containing C_60_ dyads 77 (*n* = 4, 7, 8, 9, 12), molecular packing model for dyads in the S_T_ phase and S_Q_ phase, with face-centered tetragonal fullerene packing in the 2D crystals viewed along the [001] zone and the [110] zone (blue dots indicate voids between the packed molecules and orange rectangles alkyl-tail-substituted azobenzene moieties). Adapted from ref. 468 with permission from Wiley-VCH, copyright 2018. (b) The sphere-cone molecule 78, representation of molecular packing, and TEM image of layered superlattice structure. Adapted from ref. 463 with permission from American Chemical Society, copyright 2020.

Fullerene liquid crystals (LCs) have attracted considerable attention, because they offer a combination of the excellent optoelectronic properties of fullerenes and the unique properties of liquid crystals. There are two approaches to construct fullerene LCs: the “molecular LC approach” is based on the covalent linking of fullerenes with large liquid crystal mesogens. The “supramolecular LC” method is based on supramolecular self-assembly.^462^ Using the molecular LC approach, nematic, cholesteric, smectic, and columnar phases could be achieved by introducing cholesterol and other functional groups to C_60_*via* flexible spacers or *via* a rigid “shuttlecock” geometry.^463^ However, the molecular LC approach is limited by the low content of fullerenes, which “dilutes” the optical and photophysical properties of fullerenes. The supramolecular LC approach was first developed by Nakamura and coworkers in 2002^464^ and further refined by other groups.^465^ This method relies on fullerene derivatives consisting of two parts: fullerenes and/or other PAHs provide π–π interactions between aromatic moieties, whereas and soft parts such as long alkyl chains provide vdW interactions. Both parts facilitate the assembly of highly ordered supramolecular structures with high aspect ratios, which exhibit liquid crystalline behaviour. The high fullerene content endows the fullerene-based LC with optoelectronic properties that are difficult to achieve with conventional molecular LCs.^466^ Combining fullerene LCs with 2D crystals or superlattices takes these materials properties even further.^467^ Recently, Tu, Li and coworkers employed a series of tetrablock-mimic azobenzene-containing C_60_ dyads 77 (*n* = 4, 7, 8, 9, 12) to construct supramolecular LCs.^468^ This approach allows the manipulation of smectic supramolecular LC phases by changing the alkyl tail length of dyads (Fig. 31a). These materials exhibit excellent electron mobility of *ca.* 1.5 × 10^−3^ cm^2^ V^−1^ s^−1^ due to the favourable combination of LC properties and 2D crystals. In a more recent study, well-defined superlattices were observed in supramolecular fullerene LCs by utilizing hierarchical self-assembly with a sphere-cone molecule as the building block 78. The formation of the superlattice was shown to improve the transient electron conductivity of the material (Fig. 31b).^463^

Self-assembled fullerene materials can act as sensors, catalysts, photoconductors or semiconductors in field-effect transistors.^417,418^ For instance, Li and coworkers prepared long-range M_3_N⊂C_80_ (M = Sc, Lu) single-crystal microwires by utilizing a pillar-structured template, which exhibited highly sensitive photoconductivity with a response time as low as 0.1 s, and also were employed in field-effect transistors.^469^ Molecular gels have been used as spatially confined templates for the growth of C_60_ crystals to obtain super-long crystalline C_60_ fibers and large-area 2D crystals, which were utilized as photodetectors with high performance by Liu, Fang, and coworkers.^470,471^ The dice-shaped Sc_3_N⊂C_80_ 3D crystals have been utilized as a support for the Pt-catalyzed methanol oxidation by Yang and coworkers. Catalyst performance was shown to be improved due to the larger surface area when compared with the cube shape crystals.^472^ Shrestha, Ariga and coworkers prepared hierarchically structured C_70_ cubes and corn-husk-shaped fullerene C_60_ crystals, which have been successfully applied as sensors for volatile aromatic solvents and acid vapors, respectively.^473,474^

### 6.2. Co-assembly of fullerenes and organic molecules

Fullerenes and their derivatives have been studied as building blocks to construct supramolecular (co)polymers in solution. Beside the work by Haino on helically organized fullerene arrays (see Section 4.2 or Fig. 12b),^177^ many other groups have made efforts to construct fullerene-based supramolecular polymers. For example, Langa and coworkers synthesized a fullerene-bis-Zn–porphyrin *e*-bisadduct as the monomer,^475^ which assembled into large donut structures driven by charge transfer interactions between porphyrin-bearing arms and fullerenes. This self-assembled D–A polymer possesses long-lived charge separated states upon light irradiation, specially, the lifetime of final charge-separated state is upon to 40 μs, which is relevant for single-component light harvesting devices. Sessler and coworkers employed a thiopropyl-functionalized tetrathiafulvalene-annulated calix[4]pyrrole (TTP-C[4]P) and phenyl C_61_ butyric acid (PCBA) as monomers to assemble an alternating supramolecular polymer in a 7 : 3 mixture of CHCl_3_ and CS_2_. The self-assembly is driven by the charge transfer interaction between electron rich pocket of TTF-C[4]P and fullerene and the hydrogen bonding between TTF-C[4]P and carbolic acid moiety.^476^ The supramolecular polymer could be disaggregated by addition of organic acid or electrolysis. Later, Sessler and coworkers reported an extended tetrathiafuvalene-porphyrin macrocycle, which acts as a ball-and-socket receptor for C_60_ and C_70_. This macrocycle and fullerenes assemble into 3D supramolecular organic frameworks (SOFs) in the solid state. The C_70_-based SOF exhibits remarkable electrical conductivity (*σ* = 1.3 × 10^−8^ S cm^−1^ at 298 K).^211^ These supramolecular polymers offer unique advantages over traditional covalent polymers due to the reversible nature of noncovalent interactions, such as response to the redox, pH, heating, light, or small molecules.

**Fig. 32:**
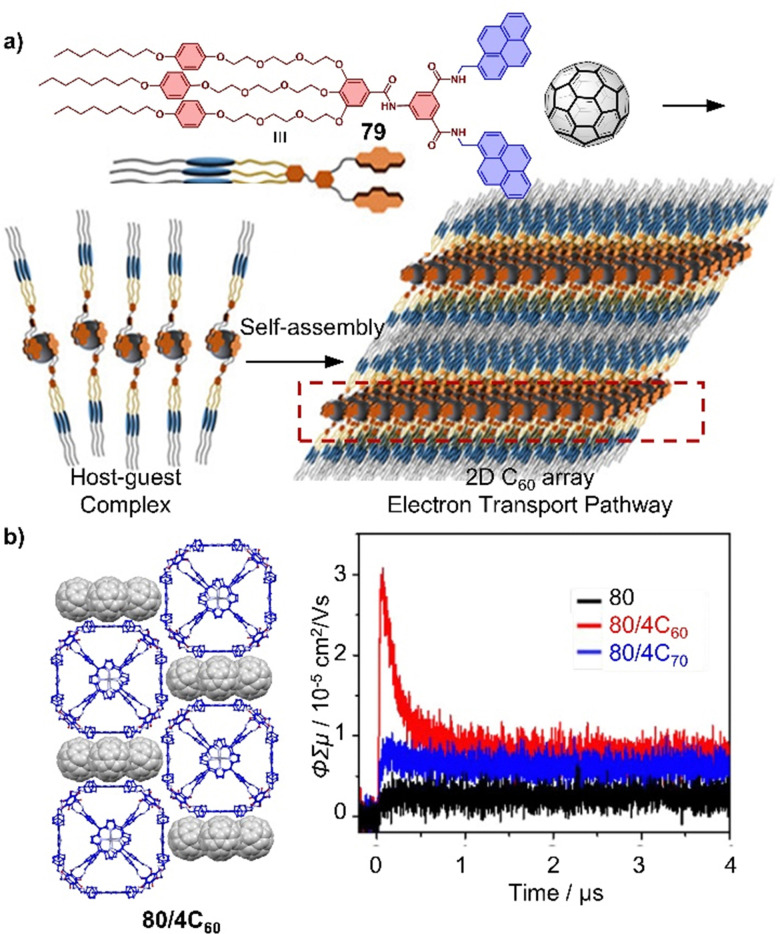
(a) Hierarchical assembly of bispyrene tweezers 79 and fullerene. Adapted from ref. 477 with permission from American Chemical Society, copyright 2019. (b) Co-assembly of porphyrin box 80 with four equivalents C_60_ and flash-photolysis time-resolved microwave conductivity of 80, 80/4C_60_, and 80/4C_70_. Adapted from ref. 482 with permission from American Chemical Society, copyright 2020.

Pristine fullerenes have been successfully co-assembled with molecules to fabricate novel functional materials. For example, Jeong, Jang, and coworkers prepared well-defined hierarchical nanostructures, consisting of a host–guest complex between pyrene-based tweezers 79 and C_60_. Due to its layered structure that comprises a 2D array of C_60_ moieties, the material exhibited high electron mobility of 1.7 × 10^−2^ cm^2^ V^−1^ s^−1^ (Fig. 32a).^477^ Although several porphyrin/fullerene supramolecular co-assemblies/crystals have been reported,^478–481^ photoinduced charge transfer between these two organic semiconductors was rarely studied, and these co-assemblies/crystals exhibited short lifetimes of charger transfer states and low charge mobility. In a co-crystal of a zinc-metalated porphyrin box 80 with C_60_/C_70_,^482^ Kim and coworkers observed a tightly packed square-planar core of four C_60_ or C_70_ surrounded by six porphyrin boxes. This tight packing pattern provides high charge mobility and allows for forming long-lived charge-separated states. Relative to crystalline box 80, a significant enhancement of photoconductivity was observed in architectures, 10-fold and 3-fold enhancement in *φΣμ* for 80/4C_60_ and 80/4C_70_, respectively. The photoconductivity of C_70_-based material is lower than C_60_-based material, which was ascribed to different electronic couplings between porphyrins and C_60_/C_70_. (Fig. 32b).

**Fig. 33:**
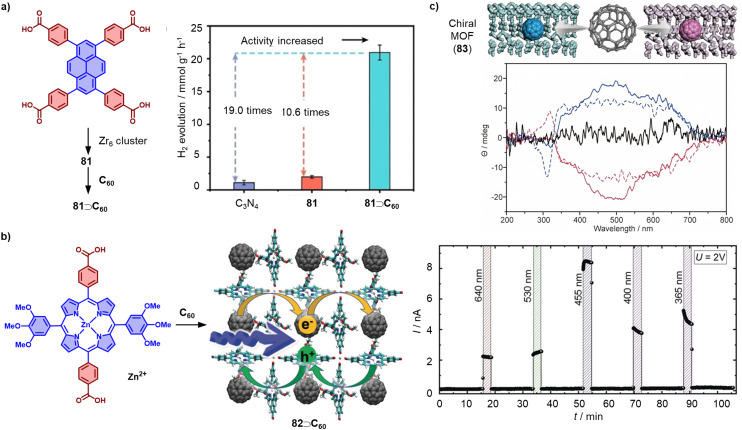
Selected encapsulations of fullerenes into MOFs to achieve: (a) improved photocatalytic hydrogen evolution, (b) improved photoconduction, (c) chirality transfer from MOF to C_60_. Adapted from ref. 489, 487 and 485 with permission from Wiley-VCH, copyright 2023, 2019, 2021 for (a), (b) and (c), respectively.

Metal–organic frameworks (MOFs) and covalent-organic frameworks (COFs) have emerged as promising materials with potential applications in a wide range of research fields.^483^ Because most MOFs or COFs have relatively large pores, the encapsulation of guests has been widely explored and fullerenes are an evident choice due to their relatively large size and optoelectronic properties.^484–494^ Berna and coworkers employed a flexible benzylic amide macrocycle attached with two carboxylic acid groups as the ligand to prepare copper(ii)- and zinc(ii)-based MOFs, which could selectively encapsulate C_60_ from a mixture of C_60_ and C_70_.^484^ The encapsulation of fullerenes was used to modulate the optoelectronic properties of MOFs due to the electronic interaction with hosts, leading to the unique properties and applications. For instance, Zhu and colleagues employed a zirconium-based MOF 81 as a host,^489^ composed of 1,3,6,8-tetrakis(4-benzoate) pyrene (TBAPy) linkers and Zr-oxo nodes, to encapsulate C_60_ (Fig. 33a). The uneven charge distribution in C_60_⊃81 provides a robust built-in electric field, which is 10.7 times higher than that in 81. Using this material, photocatalytic hydrogen evolution was found to be significantly enhanced thanks to the encapsulation of C_60_. The interplay between MOF hosts and fullerenes was also demonstrated to boost photoelectric conductivity.^486,488^ Heinke and coworkers constructed a crystalline porphyrin-based MOF (82) incorporating C_60_,^487^ providing rapid charge separation. Thanks to the efficient formation of holes and electrons, good photoconductivity was observed with an on–off photocurrent ratio of two orders of magnitude (Fig. 33b). The chirality of complex fullerene derivatives (*e.g.* certain fullerene bis-adducts) is a fascinating topic. However, imparting chirality to pristine C_60_ without installation of substituents is far from trivial. To this end, Uemura and coworkers employed a chiral MOF (83) hosting the highly symmetric C_60_ using a self-assembly strategy (Fig. 33c), of which C_60_ could be incorporated into the chiral channels of MOF.^485^ This approach can therefore endow highly symmetric, achiral compounds with chirality.

Surface co-assembly is a powerful method for creating well-organized 2D arrays that exhibit excellent performance in photovoltaic devices, sensors, and catalysts.^427^ For example, the Rabe group used hydrogen bonding to self-assemble a trimesic acid monolayer as the template for mono- or bilayers of complexes of fullerenes and oligothiophene macrocycles.^206^ Recently, Tanaka and coworkers reported the formation of a periodic monolayer of spatially separated C_60_ moieties on an Au(111) surface using carbazole-salphens or Ni-salphens containing macrocycles as fullerenes hosts. The pattern of discrete C_60_ units on the surface was thermally stable up to 200 °C under ambient pressure.^212^ In related work, Zeng and coworkers created ordered 2D C_60_ patterns on a highly oriented pyrolytic graphite (HOPG) surface using host–guest complexes of aggregation-induced emissive macrocycles and fullerenes.^495^ Rosei and coworkers employed a 2D covalent organic framework as surface-confined template to host the C_70_ moieties, imposing anchoring effects on the LC growth of C_70_ molecules, forming several fullerene-based LC mesophases, which cannot be observed under other conditions.^496^ These findings highlight the potential of fullerene-based surface co-assemblies for developing advanced materials and functional devices.

## 7. Summary and outlook

Thanks to the rapidly decreasing price of C_60_ (as low as 20 USD per gram) and the relevance of fullerenes as organic electronic materials, fullerene-based supramolecular chemistry has witnessed remarkable advances in the past decade. For instance, selective fullerene functionalization methods were developed by encapsulating fullerenes in supramolecular hosts and thus shutting down undesired reaction pathways or enhancing reactivity. With the help of such selectively modified fullerenes, supramolecular dyads and mechanically interlocked architectures have been prepared that exhibit unique optoelectronic systems. Endohedral fullerenes and open-cage fullerenes offer not only an opportunity to tune fundamental properties of fullerenes, but also a unique “playground” for the creation of functional supramolecular architectures.

Over the past decade, a large number of effective hosts for fullerenes have been synthesized, and the relevant host–guest complexes have shown significant potential in selective functionalization reactions, catalysis, the stabilization of short -lived compounds and the tuning of spin properties. Fullerenes have also been utilized as building blocks to construct covalent or non-covalent 2D/3D carbon materials, which show great potential for electron transport, conductivity, liquid crystallinity and catalysis. We expect that the recent trend towards the preparation of ordered, high-performance fullerene materials will continue and that increasing effort will be devoted towards harnessing the unique spin properties of non-conventional fullerenes, which will require further advances in both synthesis and supramolecular chemistry.

## Conflicts of interest

There are no conflicts to declare.

## Supplementary Material
